# The story of the lost twins: decoding the genetic identities of the Kumhar and Kurcha populations from the Indian subcontinent

**DOI:** 10.1186/s12863-020-00919-2

**Published:** 2020-10-22

**Authors:** Ranajit Das, Vladimir A. Ivanisenko, Anastasia A. Anashkina, Priyanka Upadhyai

**Affiliations:** 1 Yenepoya Research Centre (YRC), Yenepoya (Deemed to be University), Mangalore, Karnataka India; 2 Humanitarian Institute, Novosibirsk State University, 630090 Novosibirsk, Russia; 3 Institute of Cytology and Genetics SB RAS, Novosibirsk, Russia; 4 The Digital Health Institute, I.M. Sechenov First Moscow State Medical University (Sechenov University), Moscow, Russia; 5 Engelhardt Institute of Molecular Biology RAS, Moscow, Russia; 6 Department of Medical Genetics, Kasturba Medical College, Manipal Academy of Higher Education, Manipal, Karnataka India

**Keywords:** Kumhar, Kurchas, Indus Valley civilization, South Asian population history

## Abstract

**Background:**

The population structure of the Indian subcontinent is a tapestry of extraordinary diversity characterized by the amalgamation of autochthonous and immigrant ancestries and rigid enforcement of sociocultural stratification. Here we investigated the genetic origin and population history of the *Kumhars*, a group of people who inhabit large parts of northern India. We compared 27 previously published *Kumhar* SNP genotype data sampled from Uttar Pradesh in north India to various modern day and ancient populations.

**Results:**

Various approaches such as Principal Component Analysis (PCA), Admixture, TreeMix concurred that *Kumhars* have high ASI ancestry, minimal Steppe component and high genomic proximity to the *Kurchas*, a small and relatively little-known population found ~ 2500 km away in Kerala, south India. Given the same, biogeographical mapping using Geographic Population Structure (GPS) assigned most *Kumhar* samples in areas neighboring to those where *Kurchas* are found in south India.

**Conclusions:**

We hypothesize that the significant genomic similarity between two apparently distinct modern-day Indian populations that inhabit well separated geographical areas with no known overlapping history or links, likely alludes to their common origin during or post the decline of the Indus Valley Civilization (estimated by ALDER). Thereafter, while they dispersed towards opposite ends of the Indian subcontinent, their genomic integrity and likeness remained preserved due to endogamous social practices. Our findings illuminate the genomic history of two Indian populations, allowing a glimpse into one or few of numerous of human migrations that likely occurred across the Indian subcontinent and contributed to shape its varied and vibrant evolutionary past.

## Background

The Indian subcontinent and adjoining regions in South Asia have been a cradle for several waves of human migration during Paleolithic, Neolithic Periods, Bronze and Iron Age [1–6]. The genetic and ethnolinguistic landscape of the Indian subcontinent is remarkably heterogeneous and sculpted by the confluence of the indigenous people with immigrants that arrived into India following diverse routes [7–13]. The extant Indian gene pool is composed of largely four ancestral genetic components, namely Ancestral North Indian (ANI), Ancestral South Indian (ASI), Ancestral Tibeto-Burman (ATB), and Ancestral Austro-Asiatic (AAA) [14–16]. Recent studies dissecting the complex genetic history of South Asia suggested that a South Asia Hunter Gatherer lineage with close proximity to the present day Andamanese (AASI) admixed with individuals related to Iranian agriculturalists from Zagros mountains, Iran and West_Siberian_HG (West Siberian Hunter Gatherers) forming the Indus_Periphery gene pool, in the larger Indus valley area during the 3rd millennium BCE, and may be a vital ancestral source for the subsequent peopling of South Asia [17]. Consistent with previous evidences [11, 18] the autochthonous Indian ancestral lineages prior to their admixture with West Eurasians, likely split during eastward migration of the anatomically modern humans, out of Africa, later giving rise to AASI groups [17]. The ANI and ASI gene pools arose subsequently around ~ the 2nd millennium BCE, concurrent with the decline of the Indus Valley civilization (IVC) [19] that propelled a massive upheaval in human settlements across northern parts of the Indian subcontinent. The southward dispersal of Steppe_MLBA (later Middle to late Bronze Age Steppe) populations occurred around this time into South Asia [20–22]; it is envisioned that the Indus_Periphery related groups admixed with the Steppe_MLBA immigrants to form the ANI, while additional Indus_Periphery people migrated further south and eastward within peninsular India to mingle with AASI and formed the ASI [17]. The distinctive population structure of the Indian subcontinent is a unique amalgamation resulting from the extensive and intricate percolation of people across it for long periods together with the rigorous enforcement of sociocultural practices, such as endogamy in many groups. Interrogation of population structure, relatedness and ancestry of Indian populations provide valuable insight to not only reconstruct their evolutionary past but may also have important implications in medical genetics and understanding relevant disease biology.

Here we have investigated the population history of the *Kumhars*, a north Indian population that has likely been practicing endogamy over long periods of time, as evidenced by their Identical by descent (IBD) scores that are significantly higher than that of the Ashkenazi Jews and the Finns [23]. *Kumhars* are found throughout large parts of northern, western, and eastern India, as well as in Pakistan. The name ‘Kumhar’ is derived from the Sanskrit term ‘Kumbhakar’, which literally means earthen-pot makers alluding to their ancestral way of earning a living [24]. Interestingly, the potters from Amritsar, Punjab in north India are also known as *Kulal* or *Kalal*, a term phonetically similar to *Kulala*, a group of people from Kasaragod district of the southern Indian state of Kerala, whose traditional occupation is also pottery. The phonetic similarity between the two terms is potentially due to their common origin from the Yajurveda, an ancient Vedic Sanskrit text where potters were termed as ‘Kulals’ [25]. In this study we aimed to delineate the population history of *Kumhars* and examined their genomic similarity with other populations from the Indian subcontinent. To this end we assessed 27 previously published *Kumhar* samples, which were sampled from the north Indian state of Uttar Pradesh and compared to 2013 modern day South Asian populations [16, 23].

## Results

### Clustering of *Kumhars* in the context of other south Asian populations

**Fig. 1:**
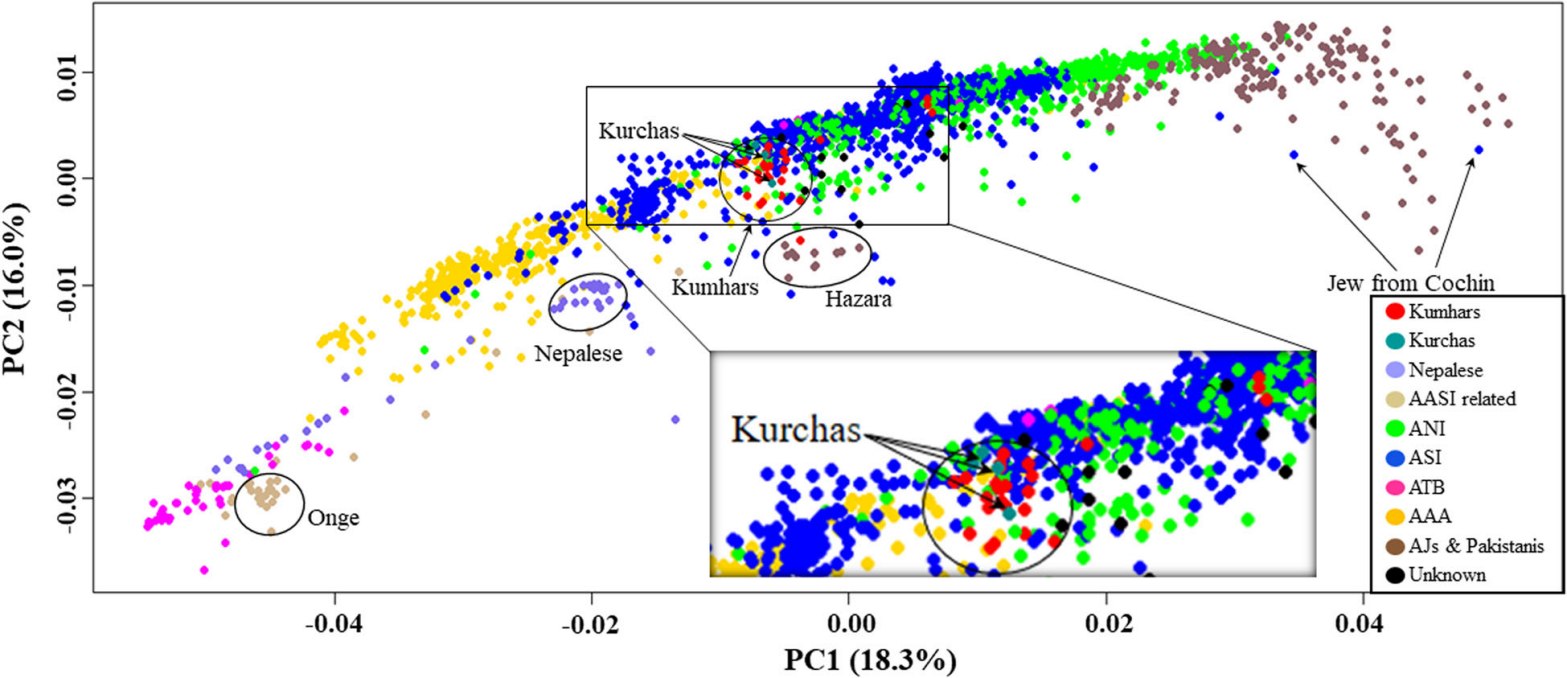
Principal Component Analysis (PCA) of South Asians. PCA plot showing genetic differentiation among South Asians. The X-axis (PC1) explained 18.3% variance while the Y-axis (PC2) explained 16% variance of the data. Notable populations are marked with circles. A more detailed population classification is shown in Additional file 1 Fig. S2. PCA was performed in PLINK v1.9 and the top four principal components (PCs) were extracted. Top two PCs (PC1 and PC2), explaining the highest variance of the data were plotted in R v3.5.1

Principal component analysis (PCA) of South Asian samples exhibited previously described [14, 26] ANI –ASI -AAA cline along the horizontal principal component (PC1) with *Ashkenazi Jews*, *Kalash* and other Pakistani populations, and *Shia Iranians* from Hyderabad clustering at one extreme of the cline, and *Juang* and other AAA populations congregating at the other extreme (Fig. 1 and Additional file 1 Fig. S2). Concurrent with our previous study [26], ASI-AAA-Ancestral Tibeto-Burman (ATB) contrast was observed along the vertical principal component (PC2) with *Juangs* clustering at one end, while *Nyshi* and other ATB populations clustered at the other end. Out of 27 *Kumhar* samples employed in this study, barring three (*stockplate_14_C2*, *stockplate_14_C4*, *stockplate_14_C6*), the remaining clustered with ASI and AAA samples, largely overlapping with tribal populations from Kerala such as the *Kurchas*. According to PCA, the only North Indian population that revealed genomic proximity to *Kumhars*, were the *Syons* from Uttarakhand. Further we note that the three above-mentioned outlier *Kumhar* samples overlapped with a population cluster that largely comprised of various non-Brahmin backward castes from Uttar Pradesh.

Weighted pairwise *F*_ST_ between *Kumhars* and 63 selected populations across India using Weir and Cockerham approach [27] implemented in PLINK v1.9 revealed the *Kumhars* to be genetically almost identical to *Kurchas* from Kerala in southern India (weighted *F*_ST_ = 0.0008) (Additional file 1 Table S1). Among the remaining populations, *Kumhars* were genetically closest to *Kurumbas* from Kerala, south India (weighted *F*_ST_ = 0.019) followed by *Vishwabrahmins* from Andhra Pradesh, south India (weighted *F*_ST_ = 0.0192) and *Chakkiliyans* from Tamil Nadu, south India (weighted *F*_ST_ = 0.0196), and farthest from largely homogeneous populations from Kerala and Tamil Nadu such as *Adiyan* (weighted *F*_ST_ = 0.044), *Paniyas* (weighted *F*_ST_ = 0.054), *Narikuruvar* (weighted *F*_ST_ = 0.055), *Pulliyar* (weighted *F*_ST_ = 0.056), *Malaikuravar* (weighted *F*_ST_ = 0.07) and *Ulladan* (weighted *F*_ST_ = 0.077). Weighted pairwise *F*_ST_ between *Kurchas* and the same 63 populations across India revealed similar results, indicating that *Kurchas* are genetically more similar to *Kumhars* than any other Indian populations including its neighboring ones (Additional file 1 Table S2).

**Fig. 2:**
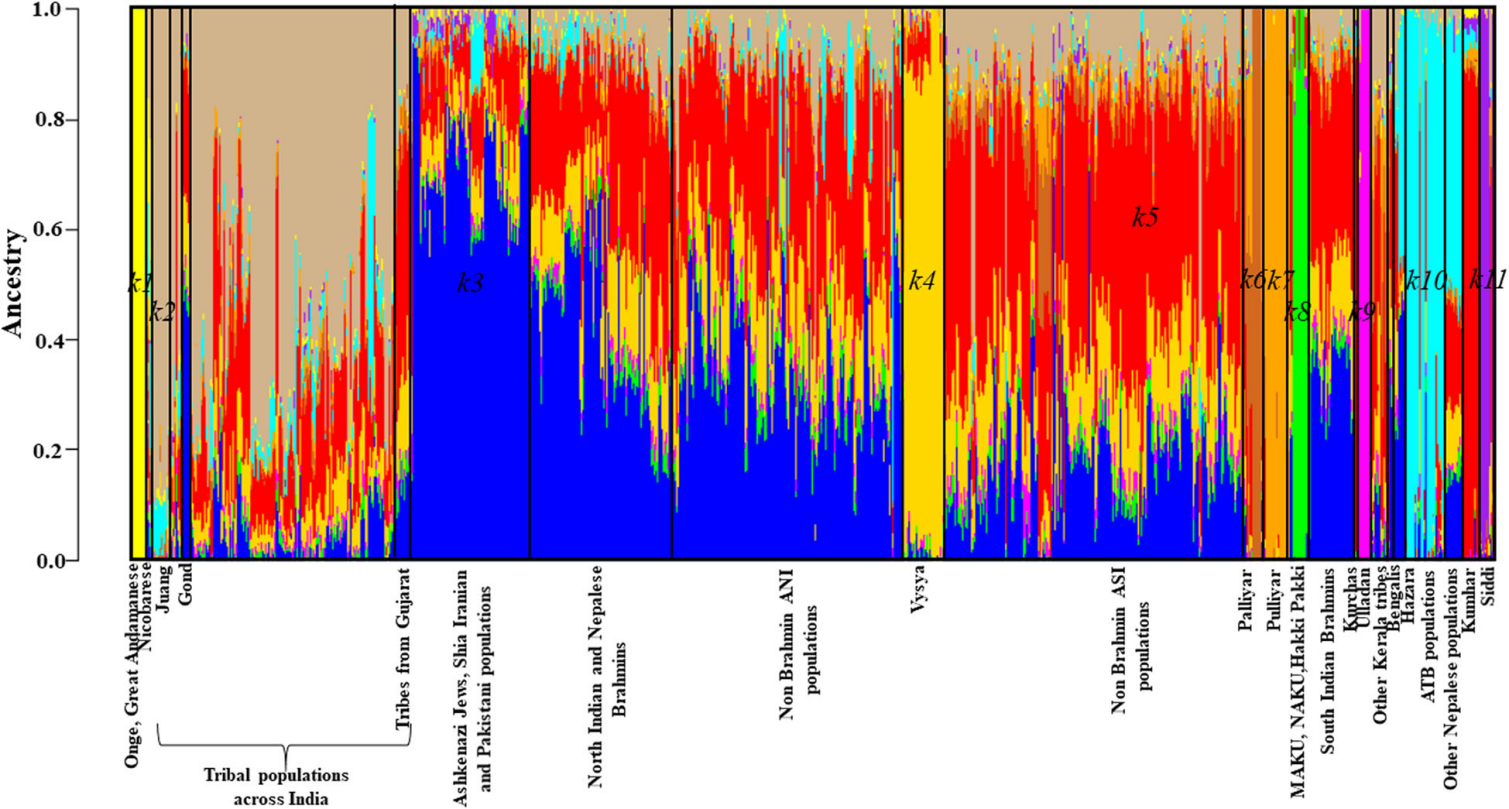
Admixture analysis of South Asians. Admixture plot showing the ancestry components of South Asian samples employed in the study. Admixture proportions were generated through an unsupervised admixture analysis at *K* = 11 using ADMIXTURE v1.3 and plotted in R v3.5.1. Each individual is represented by a vertical line partitioned into colored segments whose lengths are proportional to the contributions of the ancestral components to the genome of the individual

**Fig. 3:**
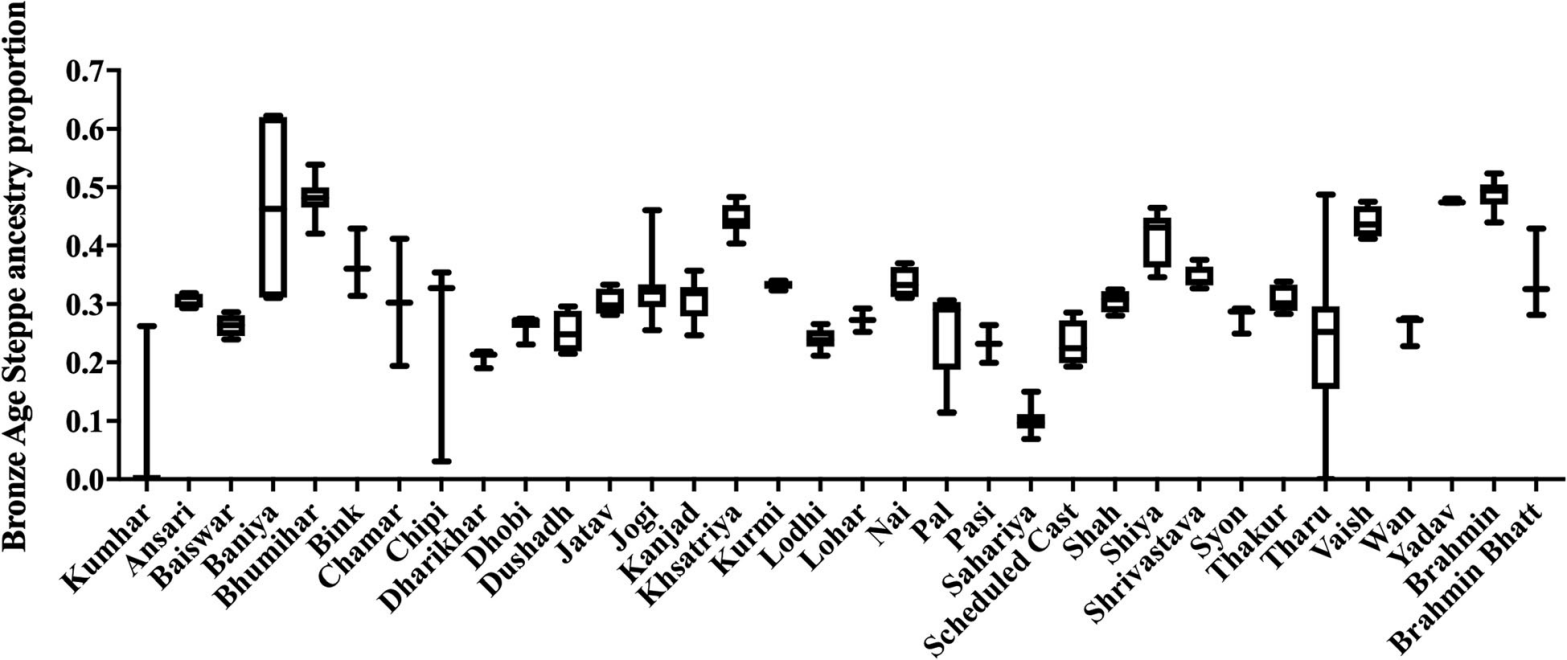
Comparison of Bronze Age Steppe Ancestry proportion among populations from Uttar Pradesh (UP) and Uttarakhand (UK). The ancestry proportions were obtained using ADMIXTURE v1.3. Multiple comparisons were performed using Tukey’s post hoc analysis implemented in GraphPad Prism v7. While Brahmin and Bhumihars contained the highest fraction of the Steppe Ancestry component (*k3*) Kumhars contained the least (Tukey’s post hoc analysis, *p*-value < 0.0001)

The genomic ancestry of all 2040 individuals present in the *Modern South Asian only* dataset was estimated using the model-based clustering algorithm ADMIXTURE v1.3 [28]. The lowest CVE was estimated for *K* = 11 (Additional file 1 Fig. S1). At *K* = 11, discernible degree of genetic admixture was observed between ANI and ASI populations (Fig. 2). *Onge* (*k1*, yellow), *Juang* (*k2*, tan), *Ashkenazi Jews* and *Shia Iranians* (*k3*, blue), *Vysya* (*k4*, gold), *Kumhar* and *Kurchas* (*k5*, red), *Palliyar* (*k6*, chocolate brown), *Pulliyar* (*k7*, orange), *Malaikuravar, Narikuruvar* and *Hakki Pakki* (*k8*, green), *Ulladan* (*k9*, magenta), ATB (*k10*, cyan), and *Siddi* (*k11*, purple) populations were assigned to distinct clusters. Congruent with previous studies [14, 16, 26], Fig. 2 revealed that most South Asians have variable fractions of blue (*k3*, likely derived from Bronze Age Steppe populations), red (*k5*, likely derived from ASI populations) and tan (*k2*, likely derived from Ancient Ancestral South Indians: AASI populations). Component *k5*, which was assigned to *Kumhars* and *Kurchas*, was found to be present in discernibly higher proportion among most non-Brahmin south Indian populations, indicating genomic similarity between *Kumhars* and ASI populations potentially linked to their common origin and admixture history. Further, the Bronze Age Steppe ancestry proportion was found to be the lowest in *Kumhars* compared to other populations from Uttar Pradesh and Uttarakhand (Fig. 3), indicating their distinct origin. We note that most *Kumhar* samples were found to have < 1% Bronze Age Steppe ancestry, except *stockplate_14_C2*, *stockplate_14_C4*, *stockplate_14_C6* (all three have 25% Steppe related ancestry), and were also found to be outliers in principal component analysis. Both PCA and ADMIXTURE analysis suggested that origin of these three *Kumhar* samples was divergent from the rest.

**Fig. 4:**
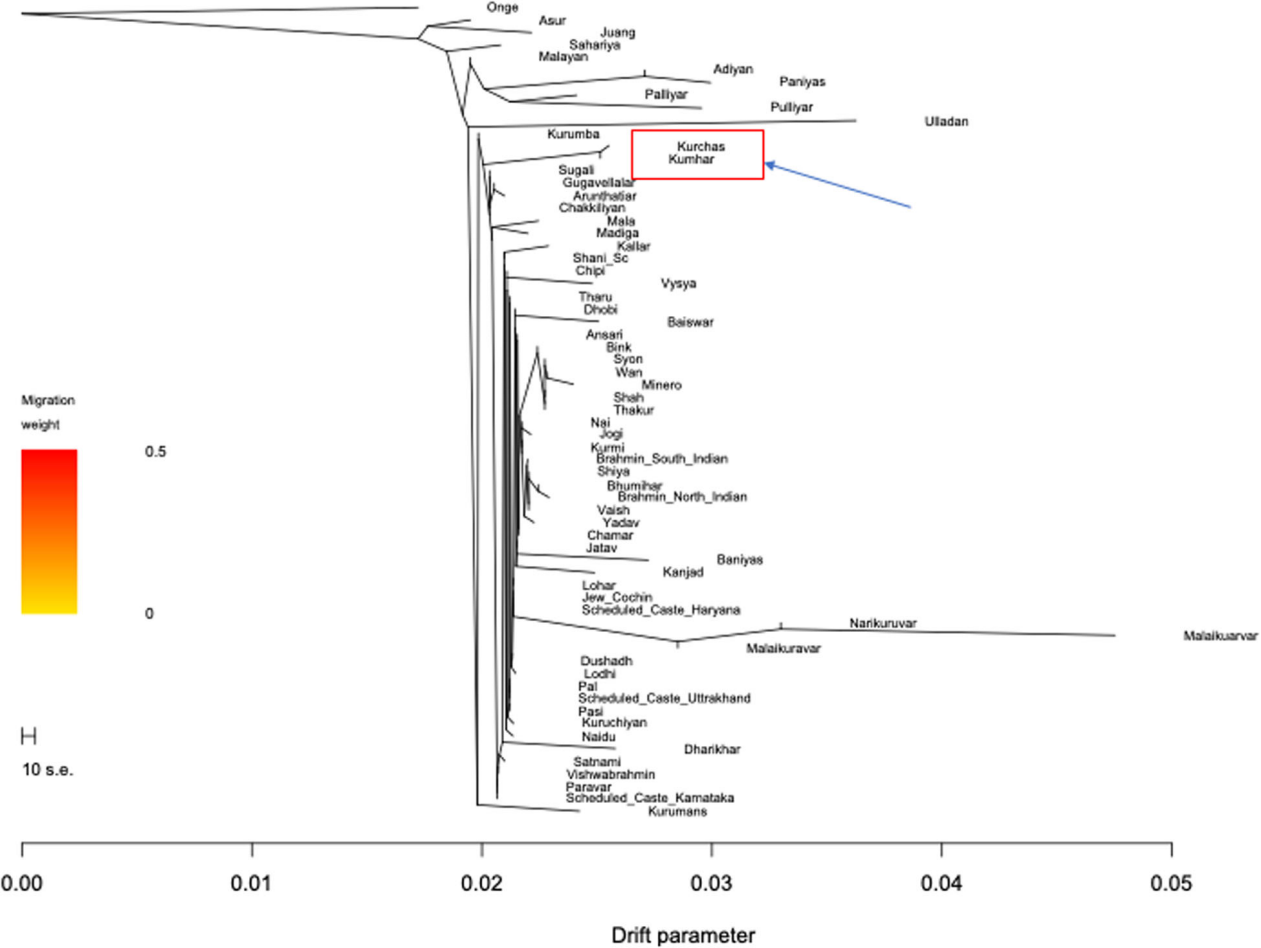
A maximum likelihood (ML) tree examining the genetic relatedness between Kumhars and selected South Asian populations. The ML tree was constructed using TreeMix v1.13. The tree was rooted using Onge, a non-African Andamanese population. Some populations are grouped together for aesthetic reason. The horizontal axis depicts the drift parameter. The scale bar shows ten times the average standard error of the entries in the sample covariance matrix. The tree confirmed the high genetic relatedness between Kumhars and Kurchas, and their genetic similarities with several South Indian populations such as Kurumba, Kuruman and Sugali

We employed TreeMix v.1.12 [29] to investigate the pattern of population splits and mixtures among selected South Asian populations. Similar to PCA, *F*_ST_ and ADMIXTURE analyses, the ML tree generated by TreeMix revealed high degree of genetic relatedness between *Kumhars* and *Kurchas*, with *Kurumba* (Kerala) and *Sugali* (Andhra Pradesh) populations as their sister groups (Fig. 4). Overall, all clustering approaches employed in this study revealed high proximity between *Kumhar* and *Kurchas* samples, and high degree of genetic similarity between *Kumhars* and several modern-day South Indian populations. On the contrary, barring the three outlier *Kumhar* samples, the remainder had very little genomic proximity towards other populations from the same geographic location in Uttar Pradesh and Uttarakhand.

We applied ALDER v.1.02 [30] to investigate the approximate time of admixture between *Kumhars* and other South Asians. 39% of the successful results in terms of LD decay included Dravidian speaking *Brahui* population from Pakistan, alongside an Austroasiatic speaking (*Oraon, Ho*) or an AASI related (*Juang*) population or a backward caste population (*Mohali, Kandha, Gond* and *Khairwar*) from north and central India (Additional file 1 Table S3). Our results indicate that the admixture event that potentially gave rise to *Kumhars* likely occurred 130–200 generations or 3640–5600 years ago, assuming a generation time of 28 years. This timeline (3600–1640 BCE) likely coincides with or after the decline of the IVC [19, 31].

### Biogeographical mapping of *Kumhar* samples employed in the study

**Fig. 5:**
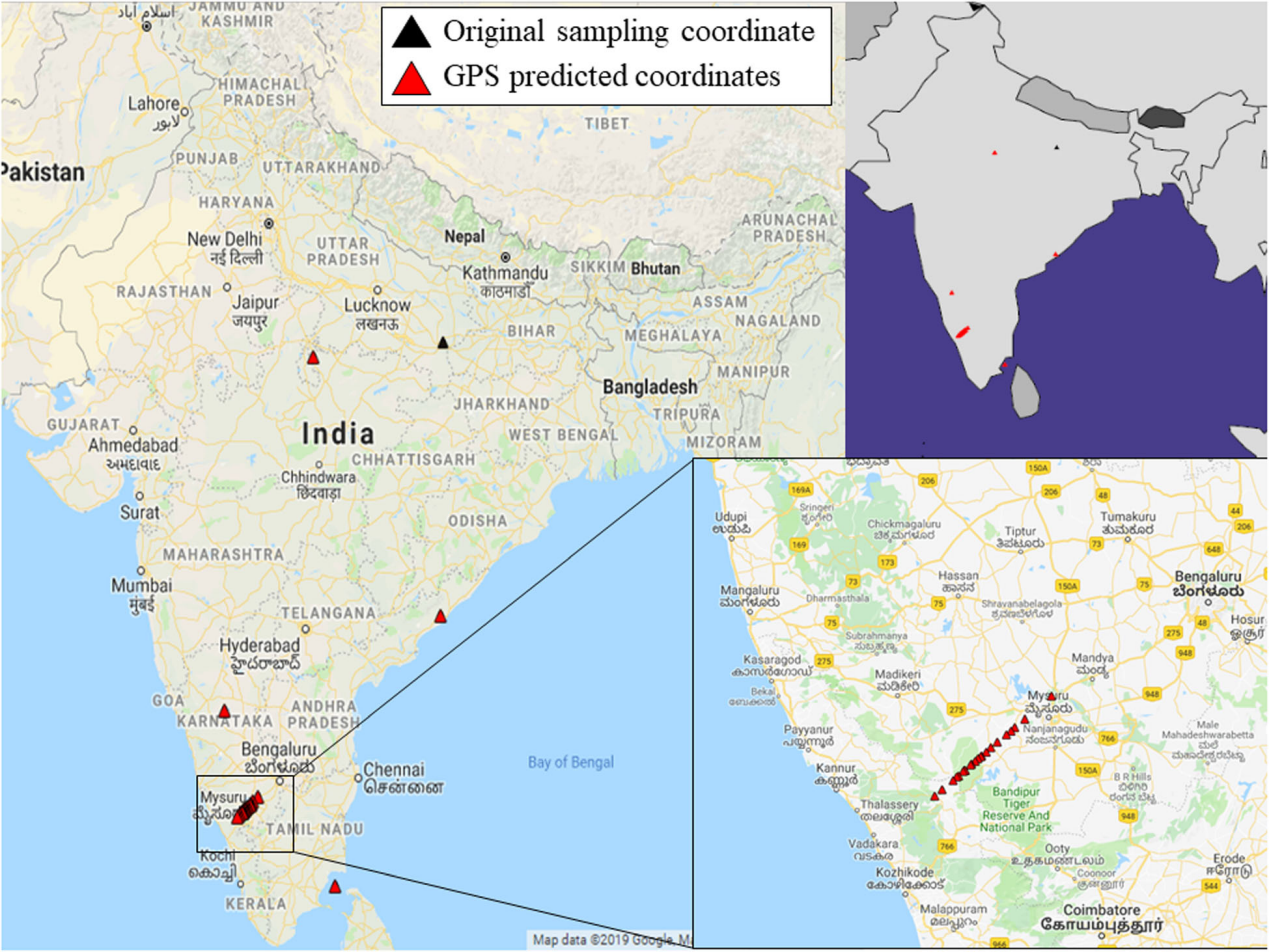
Maps depicting the GPS predicted locations of the Kumhar samples. GPS predicted coordinates were plotted in Google Maps (Google LLC) as well as using ‘rworldmap’ package implemented in R v3.5.1. The black triangle represents the original sampling location of Kumhars and the red triangles represent GPS predicted locations of Kumhars. To note, GPS localized 23 out of 27 Kumhar samples to south-western Karnataka across Western Ghats in southern India with 16 being localized at the Karnataka-Kerala border

Biogeographical mapping of *Kumhar* samples was performed using the GPS algorithm. Barring *stockplate_14_C2*, *stockplate_14_C4*, *stockplate_14_C6*, and *CCMB_PL_9_298*, GPS assigned all 23 *Kumhar* samples to south-western Karnataka across Western Ghats in southern India with 16 being localized at the Karnataka-Kerala border (Fig. 5). *Stockplate_14_C6* was positioned ~ 80 km southeast of Hubballi, northern Karnataka. *Stockplate_14_C4* was localized ~ 130 km east of Madurai, along coastal Tamil Nadu, in south India. *CCMB_PL_9_298* was assigned < 50 km west of Visakhapatnam, Andhra Pradesh, in south India. Only one *Kumhar* sample, *stockplate_14_C2* was assigned by GPS to Uttar Pradesh in north India, from where all *Kumhar* samples had been actually collected [23]; it was localized ~ 300 km southwest of Kanpur and ~ 70 km south of Jhansi. The assignment of 26 out of 27 Kumhars sampled from Uttar Pradesh, north India to South India further confirms their high genomic proximity with populations of ASI ancestry and genetic distinctness from other populations found in overlapping and neighboring areas of their geographic sampling source.

### Determination of ancestry proportions in *Kumhar*s in a global context

We used *qpAdm* [32] implemented in AdmixTools v5.1 [33] to estimate ancestry proportions in the South Asians. The *Ancient-Modern* dataset comprising of 4575 ancient and modern-day individuals worldwide was employed for this analysis. All South Asians were modelled as a combination of three source populations namely Andaman Islanders (Onge), Steppe-related (Steppe-MLBA) and Iran-Turan-related (Indus_Periphery) as *Left* (*Test, Onge, Steppe-MLBA, Indus_Periphery*) and O8 was used as the ‘*Right*’ outgroup (see Methods). Congruent with ADMIXTURE analysis, *qpAdm* analysis revealed that *Kumhars* (10.5%) are among the three populations from Uttar Pradesh, north India with low Bronze Age Steppe ancestry (Additional file 1 Table S4); the other two populations being Dharikhar (10.2%) and Sahariya tribes (0%). When we repeated the *qpAdm* analysis for *Kumhars* excluding the outlier *Kumhars* (*stockplate_14_C2*, *stockplate_14_C4* and *stockplate_14_C6*), the Steppe ancestry reduced to 9.5% and *Kumhars* emerged as the population with the second lowest Steppe ancestry after *Sahariyas*. It is noteworthy that Onge, Steppe_MLBA and Indus_Periphery ancestral component in *Kumhars*, after removal of the three outliers (49.2, 9.5 and 41.2% respectively), was found to be very similar to that in *Kurchas* (51.4, 9.3 and 39.3% respectively) and *Kurumans* from Kerala (46.2, 9.3 and 44.5% respectively), and Chakkiliyans from Tamil Nadu (47.3, 9.4 and 43.3% respectively), in south Indian reflecting the significant genomic proximity between *Kumhars* and ASI populations.

### The direction of gene flow: genetic similarities between *Kumhars* and *Kurchas*

We found positive Z-scores for all combinations of South Asian populations (X) employed in this study, indicating gene flow between *Kumhars* (W) and *Kurchas* (Y) (Additional file 1 Table S5). The positive Z-scores obtained from D-statistical analysis indicates that *Kumhars* are genetically closer to *Kurchas* than to any other Indian group employed in this study.

## Discussion

Populations from the Indian subcontinent are envisaged as a Pleistocene gene pool [10, 34–36] and are a mélange of varied indigenous and immigrant ancestries, which together with the extraordinary diversity in geographical niches and sociocultural stratification has resulted in its complex genetic history. Here we investigated the genetic origin and population history of the *Kumhars*, a group of people who traditionally worked as potters and are found over large parts of north, west and east India.

Pottery is the art of creating objects from non-metallic minerals, such as earthenware, porcelain by molding them when wet and subsequently firing them at high temperatures. It was practiced by potters referred to as Kumhars in northern, western and eastern regions of the Indian subcontinent. The origin of pottery in the Indian subcontinent can be traced back to cord-impressed style of ceramic ware from the Mesolithic period, found at the site of Lahuradewa, dating back to 7000–6000 BCE [37]. Evidences of both handmade and wheel-made forms of pottery dating back to the IVC have been obtained. The Jhukar phase of pottery corresponding to the Jhukar archaeological type-site in Sindh was coincident with urbanization in the late Harappan period [38]. This was followed by the crude handmade pottery of the Jhangar phase [38] likely reflecting a largely nomadic and pastoralist population of West Asian immigrants. The decline of the IVC and the subsequent peopling of the vast Gangetic plains in central Indian subcontinent was marked by handmade and unpainted pottery forms, such as those of the Swat grave culture and ochre colored pottery culture that further likely coincided with West Eurasian migration into the Indian subcontinent. This was followed by black and red ware and subsequently the painted grey ware cultures of pottery that likely concurred with south and eastward migration of people in the peninsular India and the formation of the ASI [39].

In India, Kumhars and their Southern counterparts such as Kulals (in Kerala), Kummara (Andhra Pradesh and Telangana), Kumbara and Kummari (Andhra Pradesh) are synonymous with pottery. We interrogated 27 previously published *Kumhar* SNP genotype data [23] and compared them to various modern day and ancient populations.

PCA revealed that except three (*stockplate_14_C2*, *stockplate_14_C4*, *stockplate_14_C6*), all *Kumhar*s congregated with those of ASI and AAA ancestries, largely overlapping with tribal populations from Kerala, in southern India, such as the *Kurchas* (Fig. 1). Similarly weighted pairwise *F*_ST_ using the Weir and Cockerham approach suggested that the *Kumhars* were genetically almost identical to *Kurchas* from Kerala, south India (weighted *F*_ST_ = 0.0008), followed by *Kurumbas* from Kerala (weighted *F*_ST_ = 0.019), *Vishwabrahmins* from Andhra Pradesh, south India (weighted *F*_ST_ = 0.0192) and *Chakkiliyans* from Tamil Nadu, south India (weighted *F*_ST_ = 0.0196) (Additional file 1 Table S1). The strong genomic proximity of *Kumhars* with *Kurchas* was further corroborated by TreeMix analysis (Fig. 4). Consistent with this, Admixture analysis also reflected a predominant ASI ancestry among the *Kumhars* that is also shared by *Kurchas* and other non-Brahmin south Indian populations (Fig. 2). For most *Kumhar* samples PCA, Admixture and *qpAdm* concurred on the presence of a minimal Steppe ancestral component (Fig. 3 and Additional file 1 Table S3).

Given the high genetic similarity between *Kumhars* and *Kurchas* it is unsurprising that biogeographical mapping of the *Kumhar*s assigned all but one sample to southern India (Fig. 5). Notably 23 *Kumhar* samples were positioned to south-western Karnataka across Western Ghats in southern India with 16 being localized at the borders of the Indian states of Karnataka and Kerala, and adjoining the geographic region of Wayanad, which is the native abode of the *Kurcha* population.

Finally using ALDER we estimated that the *Kumhar* gene pool likely arose 130–200 generations or 3640–5600 years ago coinciding with two important events that potentially occurred during and/or after the decline of IVC [19, 31]: (a) the emergence of the ASI group, which began ~ 3000 BCE during the course of the spread of West Asian domesticates into peninsular India [40] and (b) the formation of Austroasiatic speaking populations through admixture between the eastward migrating branch of out of Africa populations that arrived in South Asia ~ 3000 BCE and ancient indigenous Indian groups (AASI-related) [17, 18].

In brief, we found very little similarity between *Kumhars* and other ethnic groups from the same geographic regions in Uttar Pradesh and its adjoining state of Uttarakhand, in north India. The *Kumhars* appeared to have an overwhelming ASI ancestral component; 24 out of 27 *Kumhar*s appeared to be identical (> 98%) to a small population known as the *Kurchas* from the Wayanad district in the south Indian state of Kerala, which is approximately 2500 km south of the region from where the *Kumhar* samples were obtained. Similar to the *Kumhars* little is known regarding the *Kurcha* population and to the best of our knowledge there is no existing literature that describes any anthropological or historical connection between *Kurchas* with either the *Kumhars* or the *Kulalas*.

Here, we propose that the significant genomic similarity between two apparently distinct modern day Indian populations that correspond to well separated geographical areas separated by ~ 2500 km with no known overlapping history or links likely alludes to their common origin during or after the decline of IVC; subsequently the two populations likely migrated towards opposite ends of the Indian subcontinent but their genomic integrity was preserved owing to stringent enforcement of endogamy. Our findings illuminate the population history of two Indian groups, allowing a glimpse into one or few of numerous of human migrations that likely occurred across the Indian subcontinent and have shaped its varied and vibrant evolutionary past. Overall our findings help to reconstruct the genomic history of two Indian populations, the *Kumhars* and *Kurchas*, which despite significant geographic isolation have remained almost identical, genetically, shining light on how largescale population movements that spanned across the Indian subcontinent over extensive periods of time together with imposition of sociocultural hierarchies have contributed to its diverse evolutionary heritage.

Previous reports have indicated that the IBD scores for *Kumhars* are significantly higher than that of the Ashkenazi Jews and the Finns, consistent with social practices of consanguinity among them [23]. This is medically relevant as it predicts a high propensity for genetic disorders and consistent with this diseases such as acute intermittent porphyria are reported at higher frequencies in the *Kumhar* population [41].

### Limitations of the study

As mentioned earlier, Kumhars are distributed throughout North India and Pakistan. However, since we did not genotype/sequence any Kumhar sample and our study was completely based on previously published SNP genotype data, we were limited in terms of sample size and distribution. The same is applicable for the Kurchas. We could only employ four Kurcha samples in this study due to unavailability of Kurchas in the published datasets. It can be speculated that sampling across various parts of India can significantly improve the robustness of the study.

## Conclusions

Overall, our results reflect the high genomic similarity of *Kumhars* with various south Indian groups, including the so far little known *Kurchas*, offering some insight into the latter’s genomic history and likely predisposition to genetic disorders. It further underscores the importance of uncovering founder events among Indian populations and prioritizing them for studies dissecting genetic diseases and their underlying etiology.

## Methods

### Data sets

To generate the *Modern South Asian only* dataset we merged two previously published datasets [16, 23] using ‘mergeit’ function implemented in EIG v7.2 [42]. This dataset comprised of 2040 modern South Asian SNP genotype data, including 27 *Kumhar* samples, and corresponding to total 265 Indian ethnic groups, and assessing 91,781 single nucleotide polymorphisms (SNPs). The *Modern South Asian only* dataset was then merged with two ancient DNA datasets comprising of 294 and 362 ancient individuals, respectively (*N* = 2696) [17, 40]. Finally, this dataset was merged with 1879 modern samples [43] across the world to generate the *Ancient-Modern* dataset comprising of 4575 individuals, and assessing 91,768 SNPs. File conversions and manipulations were performed using EIG v7.2 [42] and PLINK v1.9 [44] (https://www.cog-genomics.org/plink2/).

### Genome-wide SNP data analyses

*Modern South Asian only* dataset (*N* = 2040) was used for all genome-wide SNP analyses to describe fine-scale population structure recapitulating the population history of *Kumhars*.

We calculated mean and weighted pairwise *F*_ST_ between *Kumhars* and 63 selected populations across India using the Weir and Cockerham approach [27] implemented in PLINK v1.9 [44]. PLINK estimated the fixation indices separately for all 91,781 SNPs under evaluation using –fst command alongside –family flag that enables it to group the individuals according to their family id (FID). The 63 populations comprised of all 35 available populations from Uttarakhand and Uttar Pradesh, all 10 populations from Kerala, and 18 populations from elsewhere in India.

The fine population structure of the modern South Asians was delineated using Principal Component Analysis (PCA) implemented in PLINK v1.9 [44] using –pca command. The two most informative PCs are discussed and plotted in R v3.5.1.

The genomic ancestry of all 2040 individuals was estimated using the model-based clustering algorithm ADMIXTURE v1.3 [28]. The optimum number of ancestral components (*K*) was determined by minimizing the cross-validation error (CVE) using a –cv flag to the admixture command line. The lowest CVE was estimated for *K* = 11 (Additional file 1 Fig. S1).

We constructed a maximum likelihood (ML) tree for 84 selected populations comprising of all 35 populations from Uttar Pradesh and Uttarakhand, all 10 populations from Kerala, and 39 populations across the rest of India by using TreeMix v.1.12 [29] in order to place *Kumhars* to a global context. *Onge* were used to root the ML tree.

We applied ALDER v.1.02 [30] to compute a weighted linkage disequilibrium (LD) analysis to infer the likely date of admixture, based on the exponential LD decay. We aimed to investigate the approximate time of admixture between *Kumhars* and other South Asians considering a generation time of 28 years. *Kumhars* were included as the ‘admixpop’ (admixed population) and the remaining South Asian populations present in the *Modern South Asian only* dataset were used as the ‘refpops’ (reference populations).

### Biogeographical mapping of *Kumhar* samples

Biogeographical analysis was performed using the Geographical Population Structure (GPS) algorithm, which has been successfully used to reconstruct history of several populations worldwide [45–53]. GPS correlates the admixture patterns of individuals of unknown origins using the admixture fractions (GEN file) and geographical locations or coordinates (GEO file) of reference individuals with known geographical origin. GPS converts the genetic distances between the query and the most proximal reference populations into geographic distances. Comparing the admixture proportions of the query with the reference populations, GPS extrapolates the genomic similarity of the former and infers its geographic origins using the known biogeographical information of the reference. Our test dataset comprised of the admixture fractions of *Kumhar*s. We curated the reference dataset using the rest of south Asians present in the *Modern South Asian only* dataset except *Siddis* and *Ashkenazi Jews*.

### Determination of ancestry proportions in *Kumhar*s in a global context

We used *qpAdm* [32] implemented in AdmixTools v5.1 [33] to estimate ancestry proportions in the South Asians originating from a mixture of ‘reference’ populations by utilizing shared genetic drift with a set of ‘outgroup’ populations. The *Ancient-Modern* dataset comprising of 4575 ancient and modern-day samples from across the world was employed for this analysis. In accordance with existing literature [17], three ancient samples namely Shahr-i-Soktha_MLBA2, Shahr-i-Soktha_MLBA3 and Gonur2_BA were referred to as ‘Indus_Periphery’ in *qpAdm* analysis. All South Asians were modelled as a combination of three source populations namely Andaman Islanders (*Onge*), Steppe-related (Steppe-MLBA) and Iran-Turan-related (Indus_Periphery) as *Left* (Test, Onge, Steppe-MLBA, Indus_Periphery) as already described [17]. We used a mixture of eight ancient and modern-day populations comprising of *Mabuti.DG*, *SHG*, *EHG*, *Ganj_Dareh_N*, *Anatolia_N*, *West_Siberia_N*, *Han* and *Karitiana* our ‘*Right*’ outgroup populations (O8).

### The direction of gene flow

To investigate whether Kumhar is genetically closer to Kurcha than to any other South Asian group used in this study, we employed *qpDstat* function implemented in AdmixTools v5.1 [33] in order to acquire information about the gene flow among South Asian population(s) in respect to Kumhar and Kurcha. The D-statistic was modelled as:

*Pop1 (Kumhar) Pop2 (Modern South Asian populations): Pop3 (Kurcha) Pop4 (Onge).*

Onge was used as an outgroup since it has been disconnected from the mainland populations long while ago. Here, while positive Z-scores will indicate gene flow between Kumhar and Kurcha, negative scores will indicate gene flow between other South Asian population(s) and Kurcha.

## Supplementary information

**Additional file 1.** Supplementary Material

## Data Availability

While most data are publicly available through the lab database of Dr. David Reich, Harvard University (https://reich.hms.harvard.edu/datasets) in Eigenstrat format, some were obtained from Dr. Reich’s lab through personal communication. The authors do not have the mandate to redistribute these data. Kindly contact Dr. Ranajit Das (das.ranajit@gmail.com) for details regarding the data availability.

## Abbreviations

IVC: Indus Valley Civilization
AASI: Ancient Ancestral South Indian
ANI: Ancestral North Indian
ASI: Ancestral South Indian
ATB: Ancestral Tibeto-Burman
AAA: Ancestral Austro-Asiatic
Steppe MLBA: Steppe Middle-Late Bronze Age
IBD: Identical by Descent
