# Supplementary material for: The story of the lost twins: decoding the genetic identities of the Kumhar and Kurcha populations from the Indian subcontinent
Source: BMC Genet. 2020 Oct 22;21(Suppl 1):117. doi: 10.1186/s12863-020-00919-2 (PMC7583313; doi:10.1186/s12863-020-00919-2)
Supplement: Supplementary file 1 — Additional file 1. Supplementary Material [file 12863_2020_919_MOESM1_ESM.docx]

**Supplementary Fig. S1. (A)** Table showing proportion of Cross-Validation error (CVE) in ADMIXTURE carried out for the test dataset with different values of ancestral components (*K*) employed in the admixture analysis. The CVE was used to determine the optimum number of ancestral components (*K*) supported by the data. At *K*=11 the CVE was minimized. **(B)** Plot depicting the change of CVE with increasing number of ancestral components (*K*). The optimum number of ancestral components with lowest CVE was ten (*K*=11).


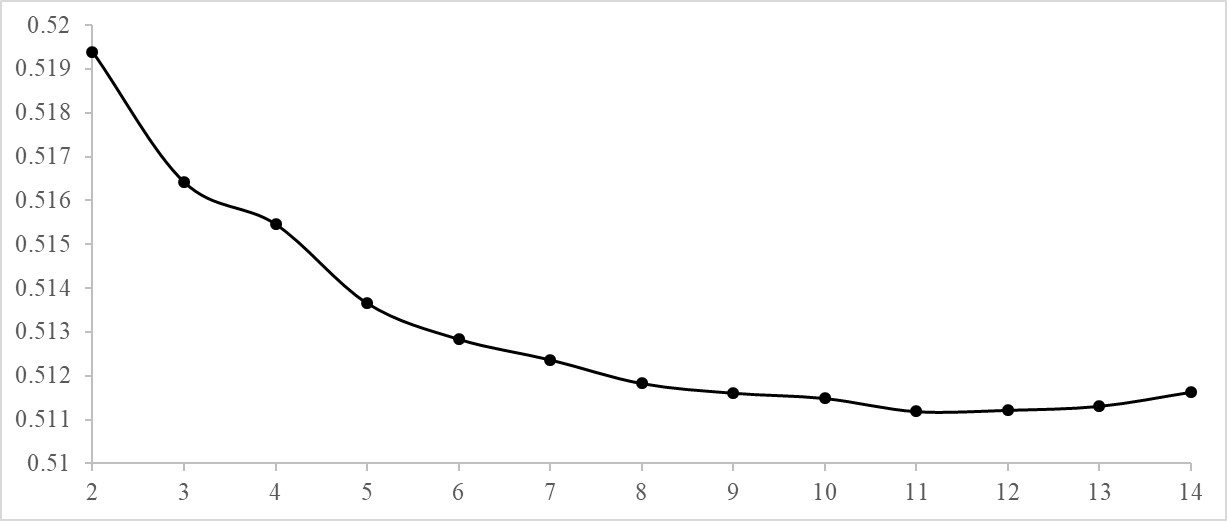
**(A) (B)**

| ***K*** | **CVE** |
| --- | --- |
| 2 | 0.51939 |
| 3 | 0.51642 |
| 4 | 0.51546 |
| 5 | 0.51366 |
| 6 | 0.51284 |
| 7 | 0.51237 |
| 8 | 0.51183 |
| 9 | 0.51161 |
| 10 | 0.51149 |
| 11 | 0.51119 |
| 12 | 0.51122 |
| 13 | 0.51131 |
| 14 | 0.51163 |

**Supplementary Fig. S2. Principal Component Analysis (PCA) of South Asian genomes.** PCA plot showing genetic differentiation among South Asian genomes. The X-axis (PC1) explained 18.3% variance while the Y-axis (PC2) explained 16% variance of the data. All notable populations are marked with circles.


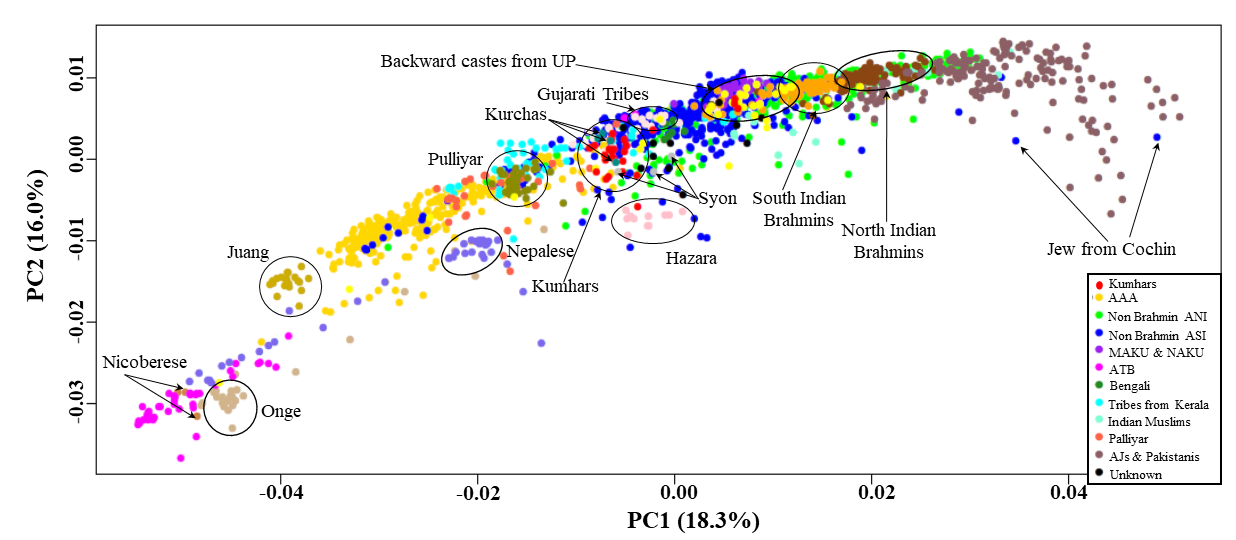


**Supplementary Fig. S3.** A maximum likelihood (ML) tree examining the genetic relatedness between Kumhars and selected South Asian populations. The ML tree was constructed using TreeMix v1.13. The tree was rooted using Onge, a non-African Andamanese population.


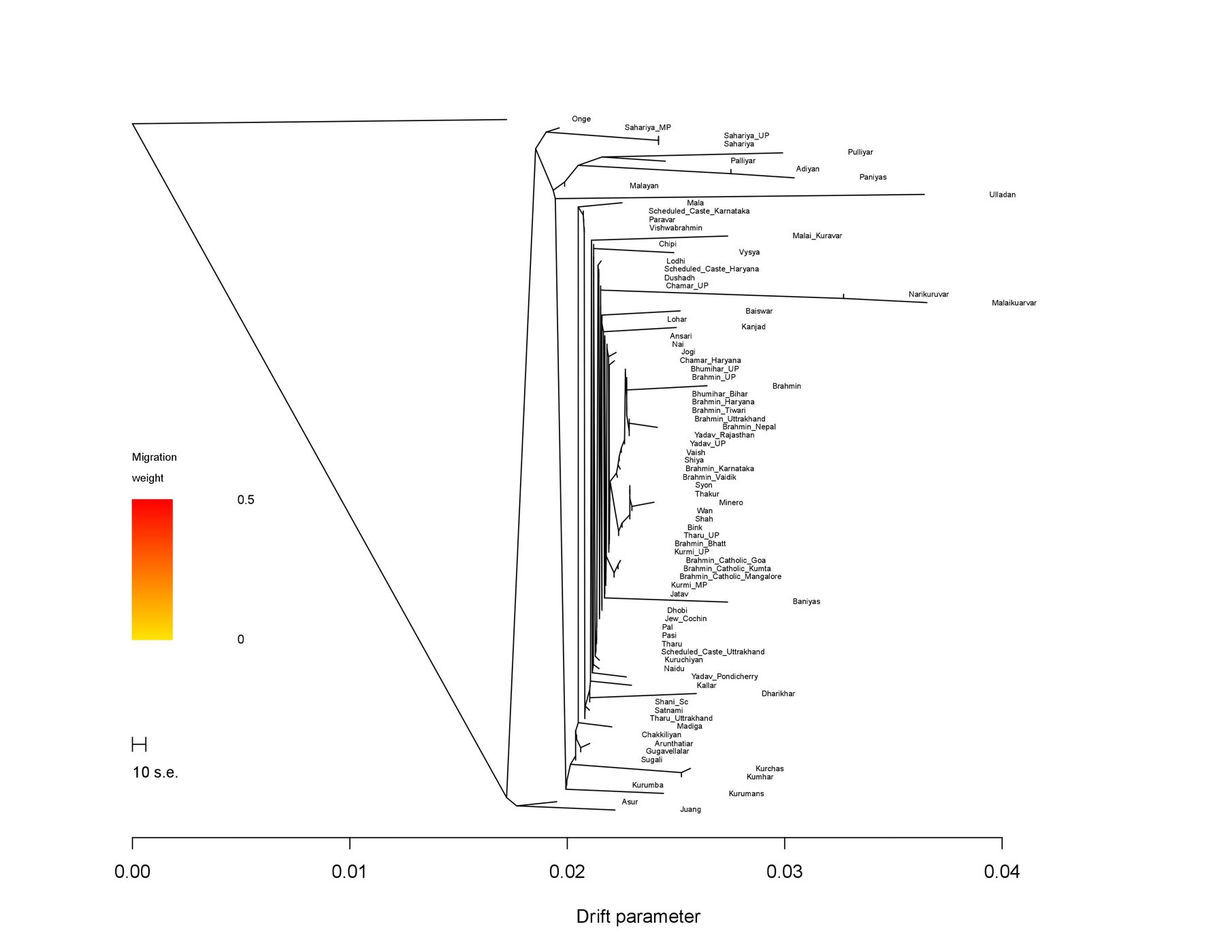


**Supplementary Table S1**: Pairwise *F*_ST_ between Kumhars and 63 selected populations across India using Weir and Cockerham approach implemented in PLINK v1.9. South, North and Central Indians are indicated by SI, NI and CI respectively. Kumhars were found to be genomically identical Kurchas from Kerala in southern India with a weighted *F*_ST_ of 0.0008.

| **Population (b)** | **State** | **N** | **Kumhar** | **N_b_** | **Mean Fst** | **Weighted global Fst** |
| --- | --- | --- | --- | --- | --- | --- |
| Adiyan | Kerala (SI) | 29 | 27 | 2 | 0.0019 | 0.0443 |
| Ansari | Uttar Pradesh (NI) | 32 | 27 | 5 | 0.0161 | 0.0220 |
| Arunthatiar | Tamil Nadu (SI) | 45 | 27 | 18 | 0.0177 | 0.0218 |
| Asur | Jharkhand (NI) | 37 | 27 | 10 | 0.0293 | 0.0375 |
| Baiswar | Uttar Pradesh (NI) | 31 | 27 | 4 | 0.0233 | 0.0368 |
| Baniya | Uttar Pradesh (NI) | 34 | 27 | 7 | 0.0322 | 0.0421 |
| Bhumihar | Uttar Pradesh (NI) | 34 | 27 | 7 | 0.0216 | 0.0269 |
| Bink | Uttarakhand (NI) | 30 | 27 | 3 | 0.0150 | 0.0297 |
| Brahmin | Uttarakhand (NI) | 33 | 27 | 6 | 0.0222 | 0.0276 |
| Brahmin | Uttar Pradesh (NI) | 42 | 27 | 15 | 0.0323 | 0.0388 |
| Brahmin_Bhatt | Uttar Pradesh (NI) | 30 | 27 | 3 | 0.0097 | 0.0241 |
| Chakkiliyan | Tamil Nadu (SI) | 43 | 27 | 16 | 0.0157 | 0.0196 |
| Chamar | Uttar Pradesh (NI) | 29 | 27 | 2 | 0.0046 | 0.0244 |
| Chipi | Uttar Pradesh (NI) | 30 | 27 | 3 | 0.0080 | 0.0208 |
| Dharikhar | Uttar Pradesh (NI) | 30 | 27 | 3 | 0.0184 | 0.0391 |
| Dhobi | Uttar Pradesh (NI) | 35 | 27 | 8 | 0.0139 | 0.0217 |
| Dushadh | Uttar Pradesh (NI) | 31 | 27 | 4 | 0.0152 | 0.0247 |
| Gugavellalar | Tamil Nadu (SI) | 30 | 27 | 3 | 0.0070 | 0.0238 |
| Jatav | Uttar Pradesh (NI) | 31 | 27 | 4 | 0.0150 | 0.0237 |
| Jew_Cochin | Kerala (SI) | 40 | 27 | 13 | 0.0174 | 0.0209 |
| Jogi | Uttar Pradesh (NI) | 35 | 27 | 8 | 0.0186 | 0.0265 |
| Kallar | Tamil Nadu (SI) | 59 | 27 | 32 | 0.0209 | 0.0254 |
| Kanjad | Uttar Pradesh (NI) | 34 | 27 | 7 | 0.0265 | 0.0343 |
| Kattunayakan | Kerala (SI) | 32 | 27 | 5 | 0.0225 | 0.0336 |
| Khsatriya | Uttar Pradesh (NI) | 42 | 27 | 15 | 0.0303 | 0.0364 |
| Kurchas | Uttar Pradesh (NI) | 31 | 27 | 4 | 0.0087 | 0.0008 |
| Kurmi | Uttar Pradesh (NI) | 32 | 27 | 5 | 0.0178 | 0.0240 |
| Kuruchiyan | Kerala (SI) | 31 | 27 | 4 | 0.0167 | 0.0239 |
| Kurumans | Kerala (SI) | 32 | 27 | 5 | 0.0242 | 0.0358 |
| Kurumba | Kerala (SI) | 36 | 27 | 9 | 0.0152 | 0.0190 |
| Lodhi | Uttar Pradesh (NI) | 38 | 27 | 11 | 0.0174 | 0.0212 |
| Lohar | Uttar Pradesh (NI) | 29 | 27 | 2 | 0.0049 | 0.0258 |
| Madiga | Andhra Pradesh (SI) | 49 | 27 | 22 | 0.0204 | 0.0247 |
| Mala | Andhra Pradesh (SI) | 56 | 27 | 29 | 0.0218 | 0.0260 |
| Malaikuravar | Tamil Nadu (SI) | 34 | 27 | 7 | 0.0515 | 0.0707 |
| Malayan | Kerala (SI) | 30 | 27 | 3 | 0.0074 | 0.0236 |
| Minero | Jammu and Kashmir (NI) | 38 | 27 | 11 | 0.0270 | 0.0325 |
| Nai | Uttar Pradesh (NI) | 31 | 27 | 4 | 0.0148 | 0.0229 |
| Narikuravar | Tamil Nadu (SI) | 34 | 27 | 7 | 0.0408 | 0.0553 |
| Pal | Uttar Pradesh (NI) | 32 | 27 | 5 | 0.0164 | 0.0226 |
| Palliyar | Tamil Nadu (SI) | 63 | 27 | 36 | 0.0291 | 0.0361 |
| Paniyas | Kerala (SI) | 32 | 27 | 5 | 0.0359 | 0.0544 |
| Paravar | Tamil Nadu (SI) | 34 | 27 | 7 | 0.0155 | 0.0199 |
| Pasi | Uttar Pradesh (NI) | 29 | 27 | 2 | 0.0054 | 0.0244 |
| Pulliyar | Tamil Nadu (SI) | 62 | 27 | 35 | 0.0460 | 0.0560 |
| Sahariya | Uttar Pradesh (NI) | 31 | 27 | 4 | 0.0177 | 0.0284 |
| Satnami | Madhya Pradesh (CI) | 36 | 27 | 9 | 0.0178 | 0.0219 |
| Scheduled_Cast | Tamil Nadu (SI) | 31 | 27 | 4 | 0.0144 | 0.0224 |
| Scheduled_Cast | Uttarakhand (NI) | 31 | 27 | 4 | 0.0136 | 0.0220 |
| Shah | Uttarakhand (NI) | 31 | 27 | 4 | 0.0215 | 0.0309 |
| Shani | Bihar (NI) | 30 | 27 | 3 | 0.0070 | 0.0213 |
| Shiya | Uttar Pradesh (NI) | 35 | 27 | 8 | 0.0209 | 0.0256 |
| Shrivastava | Uttar Pradesh (NI) | 34 | 27 | 7 | 0.0225 | 0.0282 |
| Sugali | Andhra Pradesh (SI) | 31 | 27 | 4 | 0.0129 | 0.0231 |
| Syon | Uttarakhand (NI) | 30 | 27 | 3 | 0.0143 | 0.0290 |
| Thakur | Uttarakhand (NI) | 37 | 27 | 10 | 0.0223 | 0.0265 |
| Tharu | Uttarakhand (NI) | 30 | 27 | 3 | 0.0091 | 0.0228 |
| Tharu | Uttar Pradesh (NI) | 30 | 27 | 3 | 0.0127 | 0.0263 |
| Ulladan | Kerala (SI) | 44 | 27 | 17 | 0.0583 | 0.0765 |
| Vaish | Uttar Pradesh (NI) | 31 | 27 | 4 | 0.0188 | 0.0268 |
| Vishwabrahmin | Andhra Pradesh (SI) | 39 | 27 | 12 | 0.0157 | 0.0192 |
| Wan | Uttarakhand (NI) | 30 | 27 | 3 | 0.0170 | 0.0325 |
| Yadav | Uttar Pradesh (NI) | 30 | 27 | 3 | 0.0148 | 0.0295 |

**Supplementary Table S2**: Pairwise *F*_ST_ between Kurchas and 63 selected populations across India using Weir and Cockerham approach implemented in PLINK v1.9. South, North and Central Indians are indicated by SI, NI and CI respectively.

| **Population (b)** | **State** | **N** | **Kurcha** | **N_b_** | **Mean Fst** | **Weighted global Fst** |
| --- | --- | --- | --- | --- | --- | --- |
| Adiyan | Kerala (SI) | 6 | 4 | 2 | 0.0104 | 0.0528 |
| Ansari | Uttar Pradesh (NI) | 9 | 4 | 5 | 0.0097 | 0.0260 |
| Arunthatiar | Tamil Nadu (SI) | 22 | 4 | 18 | 0.0117 | 0.0251 |
| Asur | Jharkhand (NI) | 14 | 4 | 10 | 0.0269 | 0.0442 |
| Baiswar | Uttar Pradesh (NI) | 8 | 4 | 4 | 0.0216 | 0.0433 |
| Baniya | Uttar Pradesh (NI) | 11 | 4 | 7 | 0.0304 | 0.0504 |
| Bhumihar | Uttar Pradesh (NI) | 11 | 4 | 7 | 0.0125 | 0.0296 |
| Bink | Uttarakhand (NI) | 7 | 4 | 3 | 0.0119 | 0.0337 |
| Brahmin | Uttarakhand (NI) | 10 | 4 | 6 | 0.0146 | 0.0315 |
| Brahmin | Uttar Pradesh (NI) | 19 | 4 | 15 | 0.0294 | 0.0450 |
| Brahmin_Bhatt | Uttar Pradesh (NI) | 7 | 4 | 3 | 0.0087 | 0.0306 |
| Chakkiliyan | Tamil Nadu (SI) | 20 | 4 | 16 | 0.0087 | 0.0224 |
| Chamar | Uttar Pradesh (NI) | 6 | 4 | 2 | -0.0002 | 0.0308 |
| Chipi | Uttar Pradesh (NI) | 7 | 4 | 3 | 0.0048 | 0.0244 |
| Dharikhar | Uttar Pradesh (NI) | 7 | 4 | 3 | 0.0208 | 0.0470 |
| Dhobi | Uttar Pradesh (NI) | 12 | 4 | 8 | 0.0060 | 0.0248 |
| Dushadh | Uttar Pradesh (NI) | 8 | 4 | 4 | 0.0113 | 0.0294 |
| Gugavellalar | Tamil Nadu (SI) | 7 | 4 | 3 | 0.0066 | 0.0278 |
| Jatav | Uttar Pradesh (NI) | 8 | 4 | 4 | 0.0108 | 0.0286 |
| Jew_Cochin | Kerala (SI) | 17 | 4 | 13 | 0.0078 | 0.0230 |
| Jogi | Uttar Pradesh (NI) | 12 | 4 | 8 | 0.0110 | 0.0315 |
| Kallar | Tamil Nadu (SI) | 36 | 4 | 32 | 0.0146 | 0.0292 |
| Kanjad | Uttar Pradesh (NI) | 11 | 4 | 7 | 0.0213 | 0.0393 |
| Kattunayakan | Kerala (SI) | 9 | 4 | 5 | 0.0166 | 0.0353 |
| Khsatriya | Uttar Pradesh (NI) | 19 | 4 | 15 | 0.0271 | 0.0425 |
| Kumhar | Uttar Pradesh (NI) | 31 | 4 | 27 | 0.0087 | 0.0008 |
| Kurmi | Uttar Pradesh (NI) | 9 | 4 | 5 | 0.0123 | 0.0294 |
| Kuruchiyan | Kerala (SI) | 8 | 4 | 4 | 0.0101 | 0.0262 |
| Kurumans | Kerala (SI) | 9 | 4 | 5 | 0.0213 | 0.0410 |
| Kurumba | Kerala (SI) | 13 | 4 | 9 | 0.0077 | 0.0217 |
| Lodhi | Uttar Pradesh (NI) | 15 | 4 | 11 | 0.0110 | 0.0253 |
| Lohar | Uttar Pradesh (NI) | 6 | 4 | 2 | 0.0011 | 0.0328 |
| Madiga | Andhra Pradesh (SI) | 26 | 4 | 22 | 0.0151 | 0.0286 |
| Mala | Andhra Pradesh (SI) | 33 | 4 | 29 | 0.0176 | 0.0304 |
| Malaikuravar | Tamil Nadu (SI) | 11 | 4 | 7 | 0.0563 | 0.0819 |
| Malayan | Kerala (SI) | 7 | 4 | 3 | 0.0052 | 0.0261 |
| Minero | Jammu and Kashmir (NI) | 15 | 4 | 11 | 0.0169 | 0.0343 |
| Nai | Uttar Pradesh (NI) | 8 | 4 | 4 | 0.0097 | 0.0273 |
| Narikuravar | Tamil Nadu (SI) | 11 | 4 | 7 | 0.0400 | 0.0620 |
| Pal | Uttar Pradesh (NI) | 9 | 4 | 5 | 0.0105 | 0.0271 |
| Palliyar | Tamil Nadu (SI) | 40 | 4 | 36 | 0.0228 | 0.0383 |
| Paniyas | Kerala (SI) | 9 | 4 | 5 | 0.0390 | 0.0629 |
| Paravar | Tamil Nadu (SI) | 11 | 4 | 7 | 0.0078 | 0.0221 |
| Pasi | Uttar Pradesh (NI) | 6 | 4 | 2 | 0.0020 | 0.0340 |
| Pulliyar | Tamil Nadu (SI) | 39 | 4 | 35 | 0.0479 | 0.0625 |
| Sahariya | Uttar Pradesh (NI) | 8 | 4 | 4 | 0.0145 | 0.0338 |
| Satnami | Madhya Pradesh (CI) | 13 | 4 | 9 | 0.0115 | 0.0262 |
| Scheduled_Cast | Tamil Nadu (SI) | 8 | 4 | 4 | 0.0118 | 0.0307 |
| Scheduled_Cast | Uttarakhand (NI) | 8 | 4 | 4 | 0.0088 | 0.0257 |
| Shah | Uttarakhand (NI) | 8 | 4 | 4 | 0.0152 | 0.0346 |
| Shani | Bihar (NI) | 7 | 4 | 3 | 0.0086 | 0.0299 |
| Shiya | Uttar Pradesh (NI) | 12 | 4 | 8 | 0.0109 | 0.0278 |
| Shrivastava | Uttar Pradesh (NI) | 11 | 4 | 7 | 0.0169 | 0.0338 |
| Sugali | Andhra Pradesh (SI) | 8 | 4 | 4 | 0.0062 | 0.0236 |
| Syon | Uttarakhand (NI) | 7 | 4 | 3 | 0.0104 | 0.0320 |
| Thakur | Uttarakhand (NI) | 14 | 4 | 10 | 0.0131 | 0.0291 |
| Tharu | Uttarakhand (NI) | 7 | 4 | 3 | 0.0047 | 0.0242 |
| Tharu | Uttar Pradesh (NI) | 7 | 4 | 3 | 0.0091 | 0.0301 |
| Ulladan | Kerala (SI) | 21 | 4 | 17 | 0.0708 | 0.0901 |
| Vaish | Uttar Pradesh (NI) | 8 | 4 | 4 | 0.0128 | 0.0310 |
| Vishwabrahmin | Andhra Pradesh (SI) | 16 | 4 | 12 | 0.0080 | 0.0216 |
| Wan | Uttarakhand (NI) | 7 | 4 | 3 | 0.0148 | 0.0376 |
| Yadav | Uttar Pradesh (NI) | 7 | 4 | 3 | 0.0126 | 0.0346 |

**Supplementary Table S3:** Investigation of the approximate time of admixture between Kumhars and other South Asian genomes using ALDER v1.02.

| **p-value** | **test pop** | **ref A** | **ref B** | **2-ref z-score** | **1-ref z-score A** | **1-ref z-score B** | **max decay diff %** | **2-ref decay** | **2-ref amp_exp** | **1-ref decay A** | **1-ref amp_exp A** | **1-ref decay B** | **1-ref amp_exp B** |
| --- | --- | --- | --- | --- | --- | --- | --- | --- | --- | --- | --- | --- | --- |
| 0.017 | Kumhar | Jew_Ashkenazi | Oraon | 4.91 | 3.14 | 3.33 | 131% | 129.76 +/- 22.74 | 0.00016394 +/- 0.00003337 | 47.24 +/- 15.05 | 0.00010909 +/- 0.00002191 | 27.07 +/- 8.13 | 0.00003063 +/- 0.00000507 |
| 5.10E-05 | Kumhar | Ho_Orissa | Brahui | 5.95 | 3.95 | 4.62 | 123% | 143.14 +/- 22.82 | 0.00016115 +/- 0.00002708 | 34.32 +/- 8.68 | 0.00004186 +/- 0.00000594 | 49.29 +/- 10.67 | 0.00008560 +/- 0.00001355 |
| 0.017 | Kumhar | Mohali | Brahui | 4.91 | 2.89 | 4.62 | 128% | 136.62 +/- 21.68 | 0.00013003 +/- 0.00002647 | 29.97 +/- 10.36 | 0.00004287 +/- 0.00000752 | 49.29 +/- 10.67 | 0.00008560 +/- 0.00001355 |
| 0.0024 | Kumhar | Mohali | Pandit | 5.28 | 2.89 | 4.82 | 125% | 129.73 +/- 18.88 | 0.00012408 +/- 0.00002349 | 29.97 +/- 10.36 | 0.00004287 +/- 0.00000752 | 49.39 +/- 10.25 | 0.00008288 +/- 0.00001609 |
| 0.034 | Kumhar | Juang | Kamboj | 4.77 | 4.15 | 6.09 | 111% | 119.50 +/- 23.08 | 0.00010466 +/- 0.00002192 | 41.46 +/- 10.00 | 0.00005051 +/- 0.00000876 | 34.35 +/- 5.64 | 0.00006085 +/- 0.00000729 |
| 3.90E-05 | Kumhar | Juang | Brahui | 6 | 4.15 | 4.62 | 119% | 164.20 +/- 22.99 | 0.00020970 +/- 0.00003498 | 41.46 +/- 10.00 | 0.00005051 +/- 0.00000876 | 49.29 +/- 10.67 | 0.00008560 +/- 0.00001355 |
| 0.0012 | Kumhar | Juang | Sindhi_Pakistan | 5.41 | 4.15 | 3.63 | 125% | 150.54 +/- 16.02 | 0.00013999 +/- 0.00002586 | 41.46 +/- 10.00 | 0.00005051 +/- 0.00000876 | 34.84 +/- 9.59 | 0.00005964 +/- 0.00001176 |
| 6.10E-08 | Kumhar | Juang | Dogra | 6.97 | 4.15 | 4.71 | 83% | 100.31 +/- 12.94 | 0.00007433 +/- 0.00001067 | 41.46 +/- 10.00 | 0.00005051 +/- 0.00000876 | 42.29 +/- 8.98 | 0.00006401 +/- 0.00000983 |
| 0.0076 | Kumhar | Kallar | Kamboj | 5.07 | 4.66 | 6.09 | 73% | 69.43 +/- 10.47 | 0.00001774 +/- 0.00000350 | 32.33 +/- 6.94 | 0.00004237 +/- 0.00000568 | 34.35 +/- 5.64 | 0.00006085 +/- 0.00000729 |
| 0.0022 | Kumhar | Khairwar | Brahui | 5.3 | 3.05 | 4.62 | 114% | 180.33 +/- 27.31 | 0.00020596 +/- 0.00003887 | 55.58 +/- 18.24 | 0.00006173 +/- 0.00001529 | 49.29 +/- 10.67 | 0.00008560 +/- 0.00001355 |
| 0.00076 | Kumhar | Kamboj | Oraon | 5.49 | 6.09 | 3.33 | 122% | 111.21 +/- 17.97 | 0.00006531 +/- 0.00001189 | 34.35 +/- 5.64 | 0.00006085 +/- 0.00000729 | 27.07 +/- 8.13 | 0.00003063 +/- 0.00000507 |
| 0.0071 | Kumhar | Shiya | Kandha | 5.08 | 4.21 | 4.09 | 143% | 142.10 +/- 26.53 | 0.00008387 +/- 0.00001650 | 23.46 +/- 5.57 | 0.00003944 +/- 0.00000642 | 46.85 +/- 11.45 | 0.00005837 +/- 0.00001062 |
| 2.40E-05 | Kumhar | Gond | Brahui | 6.07 | 4.35 | 4.62 | 147% | 195.64 +/- 24.20 | 0.00020814 +/- 0.00003428 | 29.86 +/- 6.87 | 0.00003877 +/- 0.00000446 | 49.29 +/- 10.67 | 0.00008560 +/- 0.00001355 |
| 9.40E-12 | Kumhar | Brahui | Oraon | 8.11 | 4.62 | 3.33 | 141% | 155.69 +/- 17.27 | 0.00015201 +/- 0.00001874 | 49.29 +/- 10.67 | 0.00008560 +/- 0.00001355 | 27.07 +/- 8.13 | 0.00003063 +/- 0.00000507 |
| 2.10E-06 | Kumhar | Brahui | Kandha | 6.46 | 4.62 | 4.09 | 96% | 133.10 +/- 19.20 | 0.00015370 +/- 0.00002381 | 49.29 +/- 10.67 | 0.00008560 +/- 0.00001355 | 46.85 +/- 11.45 | 0.00005837 +/- 0.00001062 |
| 0.00039 | Kumhar | Makrani | Bondo | 5.61 | 5.21 | 2.32 | 97% | 112.44 +/- 18.94 | 0.00015368 +/- 0.00002740 | 38.75 +/- 7.44 | 0.00007771 +/- 0.00001311 | 75.71 +/- 32.63 | 0.00008784 +/- 0.00002809 |
| 0.0061 | Kumhar | Kashmiri_Pandit | Oraon | 5.11 | 3.36 | 3.33 | 139% | 150.46 +/- 23.07 | 0.00007688 +/- 0.00001504 | 36.29 +/- 10.80 | 0.00005919 +/- 0.00001032 | 27.07 +/- 8.13 | 0.00003063 +/- 0.00000507 |
| 0.00075 | Kumhar | Shia_Iranian_Hyderabad | Kol | 5.49 | 3.76 | 3.32 | 117% | 107.95 +/- 18.66 | 0.00012740 +/- 0.00002319 | 53.94 +/- 14.36 | 0.00011039 +/- 0.00002348 | 28.35 +/- 8.55 | 0.00003687 +/- 0.00000672 |

**Supplementary Table S4**: Determination of ancestry proportions in South Asian genomes using *qpAdm* statistic implemented in AdmixTools v5.1. All South Asians were modelled as a combination of three source populations namely Andaman Islanders (Onge), Steppe-related (Steppe-MLBA) and Iran-Turan-related (Indus_Periphery) as *Left* (*Test, Onge, Steppe-MLBA, Indus_Periphery*) populations. South, North, East and Central Indians are indicated by SI, NI, EI and CI respectively.

| **Population** | **State** | **Onge** | **Steppe_MLBA** | **Indus_Periphery** |
| --- | --- | --- | --- | --- |
| Adiyan | Kerala (SI) | 0.644 | 0.077 | 0.279 |
| Ansari | Uttar Pradesh (NI) | 0.354 | 0.196 | 0.45 |
| Arunthatiar | Tamil Nadu (SI) | 0.453 | 0.062 | 0.485 |
| Asur | Jharkhand (NI) | 0.86 | -0.007 | 0.147 |
| Baiswar | Uttar Pradesh (NI) | 0.34 | 0.146 | 0.514 |
| Baniyas | Uttar Pradesh (NI) | 0.32 | 0.197 | 0.482 |
| Bhumihar_Uttar Pradesh (NI) | Uttar Pradesh (NI) | 0.227 | 0.314 | 0.459 |
| Bink | Uttarakhand (NI) | 0.447 | 0.119 | 0.434 |
| Brahmin_Bhatt | Uttar Pradesh (NI) | 0.28 | 0.214 | 0.506 |
| Brahmin_Uttar Pradesh (NI) | Uttar Pradesh (NI) | 0.223 | 0.311 | 0.466 |
| Brahmin_Uttarakhand (NI) | Uttarakhand (NI) | 0.302 | 0.278 | 0.419 |
| Chakkiliyan | Tamil Nadu (SI) | 0.473 | 0.094 | 0.433 |
| Chamar_Uttar Pradesh (NI) | Uttar Pradesh (NI) | 0.405 | 0.202 | 0.393 |
| Chipi | Uttar Pradesh (NI) | 0.461 | 0.139 | 0.4 |
| Dharikhar | Uttar Pradesh (NI) | 0.373 | 0.102 | 0.525 |
| Dhobi | Uttar Pradesh (NI) | 0.36 | 0.179 | 0.462 |
| Dushadh | Uttar Pradesh (NI) | 0.355 | 0.187 | 0.458 |
| Gugavellalar | Tamil Nadu (SI) | 0.465 | 0.062 | 0.473 |
| Jatav | Uttar Pradesh (NI) | 0.314 | 0.198 | 0.488 |
| Jew_Cochin | Kerala (SI) | 0.271 | 0.147 | 0.581 |
| Jogi | Uttar Pradesh (NI) | 0.313 | 0.2 | 0.487 |
| Juang | Orissa (EI) | 1.041 | -0.037 | -0.004 |
| Kallar | Tamil Nadu (SI) | 0.349 | 0.075 | 0.576 |
| Kanjad | Uttar Pradesh (NI) | 0.314 | 0.182 | 0.505 |
| Kattunayakan | Kerala (SI) | 0.593 | 0.041 | 0.366 |
| Kshatriya | Uttar Pradesh (NI) | 0.226 | 0.281 | 0.492 |
| Kumhar | Uttar Pradesh (NI) | 0.47 | 0.105 | 0.425 |
| Kumhar_without_Stockplate_samples | Uttar Pradesh (NI) | 0.492 | 0.095 | 0.412 |
| Kurchas | Kerala (SI) | 0.514 | 0.093 | 0.393 |
| Kurmi | Uttar Pradesh (NI) | 0.283 | 0.189 | 0.528 |
| Kuruchiyan | Kerala (SI) | 0.346 | 0.125 | 0.53 |
| Kurumans | Kerala (SI) | 0.462 | 0.093 | 0.445 |
| Kurumba | Kerala (SI) | 0.477 | 0.056 | 0.466 |
| Lodhi | Uttar Pradesh (NI) | 0.341 | 0.141 | 0.519 |
| Lohar | Uttar Pradesh (NI) | 0.3 | 0.168 | 0.532 |
| Madiga | Andhra Pradesh (SI) | 0.451 | 0.08 | 0.469 |
| Mala | Andhra Pradesh (SI) | 0.454 | 0.086 | 0.46 |
| Malaikuarvar | Tamil Nadu (SI) | 0.329 | 0.157 | 0.514 |
| Malayan | Kerala (SI) | 0.539 | 0.054 | 0.406 |
| Minero | Jammu and Kashmir (NI) | 0.454 | 0.144 | 0.403 |
| Nai | Uttar Pradesh (NI) | 0.318 | 0.223 | 0.458 |
| Narikuruvar | Tamil Nadu (SI) | 0.364 | 0.162 | 0.474 |
| Pal | Uttar Pradesh (NI) | 0.374 | 0.172 | 0.454 |
| Palliyar | Tamil Nadu (SI) | 0.605 | 0.038 | 0.357 |
| Paniyas | Kerala (SI) | 0.695 | 0.059 | 0.246 |
| Paravar | Tamil Nadu (SI) | 0.437 | 0.092 | 0.47 |
| Pasi | Uttar Pradesh (NI) | 0.396 | 0.248 | 0.357 |
| Pulliyar | Tamil Nadu (SI) | 0.577 | 0.061 | 0.362 |
| Sahariya_Madhya Pradesh | Madhya Pradesh (CI) | 0.728 | 0.073 | 0.199 |
| Sahariya_Uttar Pradesh | Uttar Pradesh (NI) | 0.665 | -0.004 | 0.339 |
| Satnami | Madhya Pradesh (CI) | 0.503 | 0.11 | 0.387 |
| Scheduled_caste_Tamil Nadu | Tamil Nadu (SI) | 0.456 | 0.071 | 0.473 |
| Scheduled_caste_Uttarakhand | Uttarakhand (NI) | 0.445 | 0.169 | 0.387 |
| Shah | Uttarakhand (NI) | 0.568 | 0.11 | 0.322 |
| Shani | Bihar (NI) | 0.448 | 0.145 | 0.407 |
| Shiya | Uttar Pradesh (NI) | 0.242 | 0.24 | 0.519 |
| Srivastava | Uttar Pradesh (NI) | 0.319 | 0.233 | 0.448 |
| Sugali | Andhra Pradesh (SI) | 0.452 | 0.065 | 0.483 |
| Syon | Uttarakhand (NI) | 0.599 | 0.103 | 0.298 |
| Thakur | Uttarakhand (NI) | 0.608 | 0.118 | 0.274 |
| Tharu_Uttar Pradesh | Uttar Pradesh (NI) | 0.645 | 0.119 | 0.235 |
| Tharu_Uttarakhand | Uttarakhand (NI) | 0.8 | 0.099 | 0.101 |
| Ulladan | Kerala (SI) | 0.583 | 0.028 | 0.39 |
| Vaish | Uttar Pradesh (NI) | 0.209 | 0.249 | 0.543 |
| Vishwabrahmin | Andhra Pradesh (SI) | 0.415 | 0.088 | 0.497 |
| Vysya | Andhra Pradesh (SI) | 0.32 | 0.064 | 0.616 |
| Wan | Uttarakhand (NI) | 0.735 | 0.078 | 0.187 |
| Yadav_Uttar Pradesh | Uttar Pradesh (NI) | 0.165 | 0.261 | 0.574 |

**Supplementary Table S5:** Test for the direction of gene flow among South Asian populations using *qpDstat* function implemented in AdmixTools v5.1. The D-statistic was modelled as: *Pop1 (Kumhar) Pop2 (Modern South Asian populations): Pop3 (Kurcha) Pop4 (Onge)*.

| **Pop1** | **Pop2** | **Pop3** | **Pop4** | **D-stat** | **Z** | **BABA** | **ABBA** | **No. of SNPs** |
| --- | --- | --- | --- | --- | --- | --- | --- | --- |
| Kumhar | Magar | Kurchas | Onge | 0.078 | 43.701 | 6139 | 5251 | 91780 |
| Kumhar | Rajbanshi | Kurchas | Onge | 0.0776 | 40.805 | 6122 | 5241 | 91780 |
| Kumhar | Sherpa | Kurchas | Onge | 0.089 | 40.657 | 6231 | 5212 | 91780 |
| Kumhar | Kharia | Kurchas | Onge | 0.0695 | 40.599 | 6020 | 5238 | 91780 |
| Kumhar | Ho_Orissa | Kurchas | Onge | 0.0671 | 40.588 | 5994 | 5241 | 91779 |
| Kumhar | Juang | Kurchas | Onge | 0.0759 | 40.587 | 6071 | 5214 | 91780 |
| Kumhar | Tibet-refugees | Kurchas | Onge | 0.0989 | 40.178 | 6315 | 5178 | 91780 |
| Kumhar | Santhal | Kurchas | Onge | 0.0662 | 39.636 | 5990 | 5246 | 91780 |
| Kumhar | Aonaga | Kurchas | Onge | 0.1035 | 38.936 | 6352 | 5160 | 91780 |
| Kumhar | Changapa | Kurchas | Onge | 0.0949 | 38.852 | 6291 | 5201 | 91780 |
| Kumhar | Kusunda | Kurchas | Onge | 0.0848 | 38.348 | 6180 | 5215 | 91780 |
| Kumhar | Hazara | Kurchas | Onge | 0.0701 | 37.847 | 6118 | 5316 | 91780 |
| Kumhar | Subba | Kurchas | Onge | 0.0935 | 37.186 | 6267 | 5195 | 91780 |
| Kumhar | Oraon | Kurchas | Onge | 0.0624 | 36.894 | 5958 | 5258 | 91780 |
| Kumhar | Kondakamari | Kurchas | Onge | 0.0625 | 36.774 | 5911 | 5215 | 90775 |
| Kumhar | Nicobarese | Kurchas | Onge | 0.1058 | 36.619 | 6370 | 5151 | 91780 |
| Kumhar | Nysha | Kurchas | Onge | 0.0981 | 36.382 | 6316 | 5187 | 91780 |
| Kumhar | Gond | Kurchas | Onge | 0.0598 | 36.281 | 5943 | 5272 | 91780 |
| Kumhar | Gond_MP | Kurchas | Onge | 0.058 | 36.16 | 5923 | 5274 | 91775 |
| Kumhar | Thakur | Kurchas | Onge | 0.0583 | 36.039 | 5982 | 5322 | 91780 |
| Kumhar | Newar | Kurchas | Onge | 0.0729 | 36.025 | 6093 | 5264 | 91780 |
| Kumhar | Bharia | Kurchas | Onge | 0.067 | 35.991 | 6001 | 5248 | 91780 |
| Kumhar | Batudi | Kurchas | Onge | 0.0715 | 35.892 | 5967 | 5171 | 90689 |
| Kumhar | Munda | Kurchas | Onge | 0.0653 | 35.807 | 5993 | 5258 | 91780 |
| Kumhar | Khairwar | Kurchas | Onge | 0.0637 | 35.237 | 5981 | 5264 | 91780 |
| Kumhar | Palliyar | Kurchas | Onge | 0.0548 | 34.943 | 5910 | 5295 | 91780 |
| Kumhar | Nagaseema | Kurchas | Onge | 0.0985 | 34.767 | 6306 | 5175 | 91777 |
| Kumhar | Mohali | Kurchas | Onge | 0.0625 | 34.521 | 5898 | 5204 | 90689 |
| Kumhar | Minero | Kurchas | Onge | 0.0573 | 34.433 | 5992 | 5342 | 91780 |
| Kumhar | Bhumij_Orissa | Kurchas | Onge | 0.0682 | 34.416 | 5993 | 5228 | 91780 |
| Kumhar | Asur | Kurchas | Onge | 0.0676 | 34.402 | 6011 | 5249 | 91780 |
| Kumhar | Kondh_TN | Kurchas | Onge | 0.0722 | 34.33 | 6035 | 5222 | 91780 |
| Kumhar | Nyshi | Kurchas | Onge | 0.0964 | 34.309 | 6308 | 5198 | 91780 |
| Kumhar | Chakehshanega | Kurchas | Onge | 0.097 | 34.092 | 6306 | 5191 | 91775 |
| Kumhar | Kandha | Kurchas | Onge | 0.0714 | 33.901 | 6041 | 5236 | 91780 |
| Kumhar | Koya | Kurchas | Onge | 0.0642 | 33.838 | 5676 | 4991 | 86808 |
| Kumhar | Ho | Kurchas | Onge | 0.0682 | 33.296 | 6003 | 5236 | 91780 |
| Kumhar | Birhor | Kurchas | Onge | 0.0664 | 33.243 | 5982 | 5237 | 91780 |
| Kumhar | Poumainaga | Kurchas | Onge | 0.1027 | 33.243 | 6341 | 5161 | 91780 |
| Kumhar | Kanwar | Kurchas | Onge | 0.0701 | 32.84 | 6022 | 5233 | 91780 |
| Kumhar | Gorait | Kurchas | Onge | 0.0576 | 32.669 | 5930 | 5285 | 91780 |
| Kumhar | Siddi | Kurchas | Onge | 0.064 | 32.113 | 6259 | 5505 | 91780 |
| Kumhar | Sahariya_MP | Kurchas | Onge | 0.0607 | 32.101 | 5966 | 5283 | 91780 |
| Kumhar | Madiga | Kurchas | Onge | 0.0477 | 31.925 | 5854 | 5320 | 91780 |
| Kumhar | Agarwal | Kurchas | Onge | 0.0442 | 31.914 | 5794 | 5304 | 90689 |
| Kumhar | Kurumba | Kurchas | Onge | 0.0525 | 31.813 | 5883 | 5296 | 91780 |
| Kumhar | Korku | Kurchas | Onge | 0.0656 | 31.748 | 5983 | 5246 | 91780 |
| Kumhar | Bhunjiya | Kurchas | Onge | 0.0658 | 31.74 | 5993 | 5253 | 91780 |
| Kumhar | Tharu | Kurchas | Onge | 0.0511 | 31.729 | 5903 | 5329 | 91780 |
| Kumhar | Bhil | Kurchas | Onge | 0.046 | 31.694 | 5848 | 5334 | 91780 |
| Kumhar | Porja | Kurchas | Onge | 0.0752 | 31.678 | 6069 | 5221 | 91777 |
| Kumhar | Khasi | Kurchas | Onge | 0.0885 | 31.619 | 6214 | 5204 | 91780 |
| Kumhar | Mala | Kurchas | Onge | 0.0456 | 31.478 | 5837 | 5328 | 91780 |
| Kumhar | Lohra | Kurchas | Onge | 0.0623 | 31.414 | 5956 | 5258 | 91780 |
| Kumhar | Bhumij | Kurchas | Onge | 0.0661 | 31.253 | 5988 | 5245 | 91780 |
| Kumhar | Bhumij_Jharkhand | Kurchas | Onge | 0.0675 | 31.159 | 6004 | 5244 | 91780 |
| Kumhar | Chakkiliyan | Kurchas | Onge | 0.047 | 31.119 | 5853 | 5327 | 91780 |
| Kumhar | Burusho | Kurchas | Onge | 0.0477 | 31.097 | 5928 | 5387 | 91780 |
| Kumhar | Satnami | Kurchas | Onge | 0.0517 | 31.044 | 5890 | 5311 | 91780 |
| Kumhar | Sahariya | Kurchas | Onge | 0.0632 | 31.032 | 5981 | 5270 | 91780 |
| Kumhar | Kallar | Kurchas | Onge | 0.0462 | 31.018 | 5856 | 5339 | 91780 |
| Kumhar | Pulliyar | Kurchas | Onge | 0.0557 | 30.834 | 5612 | 5020 | 86808 |
| Kumhar | Irula | Kurchas | Onge | 0.0534 | 30.777 | 5895 | 5297 | 91780 |
| Kumhar | Sikh_Jatt | Kurchas | Onge | 0.0453 | 30.714 | 5825 | 5321 | 90689 |
| Kumhar | Nadar | Kurchas | Onge | 0.0471 | 30.602 | 5584 | 5081 | 86808 |
| Kumhar | Manjhi_Jharkhand | Kurchas | Onge | 0.0629 | 30.58 | 5964 | 5259 | 91780 |
| Kumhar | Jew_Cochin | Kurchas | Onge | 0.0465 | 30.541 | 5886 | 5363 | 91780 |
| Kumhar | Arunthatiar | Kurchas | Onge | 0.047 | 30.451 | 5848 | 5322 | 91780 |
| Kumhar | Hallaki | Kurchas | Onge | 0.0469 | 30.033 | 5859 | 5334 | 91780 |
| Kumhar | Brahmin_Tiwari | Kurchas | Onge | 0.0452 | 29.375 | 5884 | 5375 | 91780 |
| Kumhar | Parhaiya | Kurchas | Onge | 0.0619 | 29.361 | 5939 | 5247 | 91780 |
| Kumhar | Handigodu | Kurchas | Onge | 0.0487 | 29.34 | 5800 | 5261 | 90689 |
| Kumhar | Kol | Kurchas | Onge | 0.0578 | 29.294 | 5940 | 5291 | 91780 |
| Kumhar | Brahmin_Vaidik | Kurchas | Onge | 0.0431 | 29.15 | 5792 | 5313 | 90689 |
| Kumhar | Yadav_Pondicherry | Kurchas | Onge | 0.0454 | 28.565 | 5845 | 5337 | 91780 |
| Kumhar | Lodhi | Kurchas | Onge | 0.045 | 28.516 | 5840 | 5338 | 91780 |
| Kumhar | Shah | Kurchas | Onge | 0.0597 | 28.413 | 5995 | 5320 | 91780 |
| Kumhar | Brahmin_Catholic_Goa | Kurchas | Onge | 0.044 | 28.326 | 5864 | 5370 | 91780 |
| Kumhar | Naidu | Kurchas | Onge | 0.0455 | 28.237 | 5845 | 5336 | 91780 |
| Kumhar | Vysya | Kurchas | Onge | 0.0424 | 28.104 | 5822 | 5349 | 91780 |
| Kumhar | Vishwabrahmin | Kurchas | Onge | 0.0446 | 28.011 | 5841 | 5342 | 91780 |
| Kumhar | Tharu_Uttrakhand | Kurchas | Onge | 0.0608 | 27.857 | 5981 | 5295 | 91780 |
| Kumhar | Bondo | Kurchas | Onge | 0.0733 | 27.795 | 6049 | 5223 | 91780 |
| Kumhar | Bengali | Kurchas | Onge | 0.0506 | 27.694 | 5894 | 5327 | 91780 |
| Kumhar | Kashmiri_Pandit | Kurchas | Onge | 0.045 | 27.667 | 5892 | 5385 | 91780 |
| Kumhar | Kashmiri_Pandit | Kurchas | Onge | 0.045 | 27.667 | 5892 | 5385 | 91780 |
| Kumhar | Mawasi | Kurchas | Onge | 0.067 | 27.625 | 5997 | 5243 | 91780 |
| Kumhar | Hojo | Kurchas | Onge | 0.0677 | 27.6 | 5938 | 5185 | 90689 |
| Kumhar | Adi_Dravider | Kurchas | Onge | 0.0459 | 27.593 | 5783 | 5276 | 90689 |
| Kumhar | Brahmin_Catholic_Kumta | Kurchas | Onge | 0.0446 | 27.505 | 5859 | 5359 | 91780 |
| Kumhar | Muslim_Karnataka | Kurchas | Onge | 0.0504 | 27.381 | 5615 | 5076 | 86808 |
| Kumhar | Shiya | Kurchas | Onge | 0.0461 | 27.357 | 5894 | 5375 | 91779 |
| Kumhar | Panta_Kapu | Kurchas | Onge | 0.0423 | 27.236 | 5773 | 5305 | 90689 |
| Kumhar | Kshatriya | Kurchas | Onge | 0.0446 | 27.048 | 5878 | 5377 | 91780 |
| Kumhar | Ghasia | Kurchas | Onge | 0.0546 | 26.968 | 5925 | 5311 | 91780 |
| Kumhar | Gond_Chattisgarh | Kurchas | Onge | 0.061 | 26.848 | 5949 | 5265 | 91780 |
| Kumhar | Dhobi | Kurchas | Onge | 0.0447 | 26.768 | 5847 | 5347 | 91702 |
| Kumhar | Punjabi | Kurchas | Onge | 0.0457 | 26.469 | 5867 | 5354 | 91780 |
| Kumhar | Muslim_Kashmiri | Kurchas | Onge | 0.0444 | 26.435 | 5893 | 5391 | 91780 |
| Kumhar | Kamboj | Kurchas | Onge | 0.0427 | 26.371 | 5900 | 5417 | 91780 |
| Kumhar | Chaurasia | Kurchas | Onge | 0.0461 | 26.286 | 5576 | 5085 | 86808 |
| Kumhar | Chauhan | Kurchas | Onge | 0.0506 | 26.172 | 5882 | 5316 | 91780 |
| Kumhar | Halba | Kurchas | Onge | 0.0593 | 26.114 | 5928 | 5264 | 91780 |
| Kumhar | Mohli | Kurchas | Onge | 0.0569 | 26.066 | 5925 | 5287 | 91780 |
| Kumhar | Kotwalia | Kurchas | Onge | 0.05 | 25.993 | 5864 | 5306 | 91780 |
| Kumhar | Kolcha | Kurchas | Onge | 0.0506 | 25.985 | 5873 | 5307 | 91780 |
| Kumhar | Syon | Kurchas | Onge | 0.058 | 25.913 | 5975 | 5319 | 91780 |
| Kumhar | Paravar | Kurchas | Onge | 0.0467 | 25.846 | 5855 | 5333 | 91780 |
| Kumhar | Lambadi | Kurchas | Onge | 0.0472 | 25.669 | 5880 | 5351 | 91780 |
| Kumhar | Pal | Kurchas | Onge | 0.0482 | 25.635 | 5870 | 5330 | 91780 |
| Kumhar | Didayi | Kurchas | Onge | 0.0686 | 25.585 | 5999 | 5228 | 91780 |
| Kumhar | Jogi | Kurchas | Onge | 0.0456 | 25.538 | 5860 | 5349 | 91588 |
| Kumhar | Scheduled_Caste_Karnataka | Kurchas | Onge | 0.0463 | 25.513 | 5841 | 5324 | 91780 |
| Kumhar | Yerukali | Kurchas | Onge | 0.0464 | 25.495 | 5854 | 5334 | 91780 |
| Kumhar | Minicoy | Kurchas | Onge | 0.0494 | 25.427 | 5892 | 5338 | 91780 |
| Kumhar | Brahmin_Uttrakhand | Kurchas | Onge | 0.0473 | 25.271 | 5906 | 5372 | 91780 |
| Kumhar | Paniyas | Kurchas | Onge | 0.0588 | 25.249 | 5919 | 5262 | 91780 |
| Kumhar | Sindhi_Pakistan | Kurchas | Onge | 0.0417 | 25.143 | 5875 | 5404 | 91780 |
| Kumhar | Siddi_Karnataka | Kurchas | Onge | 0.0611 | 25.06 | 6209 | 5494 | 91780 |
| Kumhar | Pathan | Kurchas | Onge | 0.0426 | 25.029 | 5897 | 5415 | 91780 |
| Kumhar | Rathwa | Kurchas | Onge | 0.0472 | 25.02 | 5850 | 5323 | 91780 |
| Kumhar | Malayan | Kurchas | Onge | 0.0535 | 25.016 | 5892 | 5294 | 91780 |
| Kumhar | Reddy_Telangana | Kurchas | Onge | 0.0438 | 24.909 | 5835 | 5346 | 91780 |
| Kumhar | Kattunayakkan | Kurchas | Onge | 0.0521 | 24.896 | 5899 | 5315 | 91780 |
| Kumhar | Gounder | Kurchas | Onge | 0.0438 | 24.777 | 5845 | 5355 | 91780 |
| Kumhar | Brahmin_Catholic_Mangalore | Kurchas | Onge | 0.0447 | 24.755 | 5854 | 5353 | 91780 |
| Kumhar | Ulladan | Kurchas | Onge | 0.0524 | 24.736 | 5872 | 5287 | 91778 |
| Kumhar | Oswal_Jain | Kurchas | Onge | 0.0482 | 24.721 | 5606 | 5090 | 86807 |
| Kumhar | Manjhi_MP | Kurchas | Onge | 0.0491 | 24.7 | 5887 | 5336 | 91780 |
| Kumhar | Tharu_UP | Kurchas | Onge | 0.0584 | 24.678 | 5978 | 5318 | 91777 |
| Kumhar | Brahmin | Kurchas | Onge | 0.043 | 24.44 | 5871 | 5386 | 91780 |
| Kumhar | Panika_Jharkhand | Kurchas | Onge | 0.0534 | 24.437 | 5895 | 5298 | 91780 |
| Kumhar | Lodi | Kurchas | Onge | 0.0449 | 24.423 | 5837 | 5335 | 91780 |
| Kumhar | Bhumihar_Bihar | Kurchas | Onge | 0.0437 | 24.285 | 5877 | 5385 | 91780 |
| Kumhar | Kunabi | Kurchas | Onge | 0.0452 | 24.205 | 5845 | 5339 | 91780 |
| Kumhar | Chaudhary | Kurchas | Onge | 0.0478 | 24.163 | 5852 | 5318 | 91780 |
| Kumhar | Wan | Kurchas | Onge | 0.0613 | 24.143 | 6003 | 5310 | 91780 |
| Kumhar | Srivastava | Kurchas | Onge | 0.0432 | 24.127 | 5846 | 5362 | 91780 |
| Kumhar | Kamsali | Kurchas | Onge | 0.046 | 23.988 | 5862 | 5346 | 91780 |
| Kumhar | Adi-Dravider | Kurchas | Onge | 0.0464 | 23.875 | 5843 | 5325 | 91780 |
| Kumhar | Hindumalayali | Kurchas | Onge | 0.0486 | 23.84 | 5858 | 5315 | 91780 |
| Kumhar | Kurumans | Kurchas | Onge | 0.0477 | 23.769 | 5868 | 5334 | 91780 |
| Kumhar | Jews | Kurchas | Onge | 0.0472 | 23.743 | 5894 | 5362 | 91780 |
| Kumhar | Warli | Kurchas | Onge | 0.0488 | 23.715 | 5855 | 5310 | 91780 |
| Kumhar | Kanjad | Kurchas | Onge | 0.0452 | 23.713 | 5861 | 5354 | 91780 |
| Kumhar | Kurmi_UP | Kurchas | Onge | 0.0444 | 23.686 | 5856 | 5358 | 91780 |
| Kumhar | Koli | Kurchas | Onge | 0.0486 | 23.657 | 5855 | 5312 | 91780 |
| Kumhar | Garasia | Kurchas | Onge | 0.0465 | 23.577 | 5847 | 5328 | 91780 |
| Kumhar | Scheduled_Caste_Uttrakhand | Kurchas | Onge | 0.0474 | 23.529 | 5870 | 5338 | 91780 |
| Kumhar | Sindhi_MP | Kurchas | Onge | 0.0454 | 23.51 | 5856 | 5347 | 91780 |
| Kumhar | Malli | Kurchas | Onge | 0.0463 | 23.504 | 5873 | 5352 | 91780 |
| Kumhar | Panika_MP | Kurchas | Onge | 0.0546 | 23.44 | 5906 | 5294 | 91716 |
| Kumhar | Sahariya_UP | Kurchas | Onge | 0.0619 | 23.43 | 5976 | 5280 | 91747 |
| Kumhar | Yanidi | Kurchas | Onge | 0.0499 | 23.3 | 5818 | 5265 | 90689 |
| Kumhar | Chenchu | Kurchas | Onge | 0.053 | 23.28 | 5897 | 5303 | 91780 |
| Kumhar | Patel | Kurchas | Onge | 0.0431 | 23.276 | 5843 | 5361 | 91780 |
| Kumhar | Jatav | Kurchas | Onge | 0.0468 | 23.234 | 5865 | 5340 | 91780 |
| Kumhar | Vaish | Kurchas | Onge | 0.0455 | 23.181 | 5885 | 5373 | 91780 |
| Kumhar | Balochi | Kurchas | Onge | 0.0419 | 23.157 | 5911 | 5435 | 91780 |
| Kumhar | Bhumihar_UP | Kurchas | Onge | 0.0422 | 23.145 | 5861 | 5387 | 91682 |
| Kumhar | Baiga | Kurchas | Onge | 0.0644 | 23.053 | 5982 | 5258 | 91780 |
| Kumhar | Ansari | Kurchas | Onge | 0.0462 | 23.033 | 5864 | 5347 | 91780 |
| Kumhar | Yadav_Rajasthan | Kurchas | Onge | 0.0447 | 23.022 | 5883 | 5379 | 91780 |
| Kumhar | Korava | Kurchas | Onge | 0.0464 | 22.981 | 5857 | 5337 | 91780 |
| Kumhar | Kshatriya_Durgvanshi | Kurchas | Onge | 0.0463 | 22.907 | 5888 | 5366 | 91780 |
| Kumhar | Gadaba | Kurchas | Onge | 0.078 | 22.906 | 5985 | 5119 | 90700 |
| Kumhar | GujaratiC | Kurchas | Onge | 0.0443 | 22.894 | 5867 | 5369 | 91780 |
| Kumhar | Havik | Kurchas | Onge | 0.044 | 22.855 | 5864 | 5369 | 91780 |
| Kumhar | Meena | Kurchas | Onge | 0.0431 | 22.845 | 5854 | 5370 | 91780 |
| Kumhar | Brahui | Kurchas | Onge | 0.0407 | 22.818 | 5901 | 5440 | 91780 |
| Kumhar | Muthuliar | Kurchas | Onge | 0.0454 | 22.703 | 5850 | 5342 | 91780 |
| Kumhar | Kuruba | Kurchas | Onge | 0.0448 | 22.6 | 5847 | 5346 | 91780 |
| Kumhar | Sugali | Kurchas | Onge | 0.0468 | 22.565 | 5784 | 5267 | 90689 |
| Kumhar | Kshatriya_Aquikula | Kurchas | Onge | 0.047 | 22.531 | 5865 | 5338 | 91780 |
| Kumhar | Narikuruvar | Kurchas | Onge | 0.0466 | 22.511 | 5856 | 5335 | 91780 |
| Kumhar | Bink | Kurchas | Onge | 0.0511 | 22.416 | 5936 | 5358 | 91780 |
| Kumhar | Gaud_Telangana | Kurchas | Onge | 0.0458 | 22.394 | 5845 | 5333 | 91780 |
| Kumhar | Baniyas | Kurchas | Onge | 0.0438 | 22.358 | 5852 | 5361 | 91780 |
| Kumhar | Dogra | Kurchas | Onge | 0.0443 | 22.275 | 5889 | 5390 | 91780 |
| Kumhar | Brahmin_UP | Kurchas | Onge | 0.0444 | 22.226 | 5874 | 5375 | 91780 |
| Kumhar | Ediga | Kurchas | Onge | 0.0478 | 22.215 | 5575 | 5066 | 86808 |
| Kumhar | Gond_Raj | Kurchas | Onge | 0.0603 | 22.146 | 5652 | 5009 | 86789 |
| Kumhar | Nai | Kurchas | Onge | 0.045 | 22.109 | 5857 | 5353 | 91780 |
| Kumhar | Kondh_AP | Kurchas | Onge | 0.0762 | 22.085 | 5990 | 5142 | 90466 |
| Kumhar | Dudhekula | Kurchas | Onge | 0.0465 | 22.082 | 5795 | 5281 | 90689 |
| Kumhar | Hakki_Pikki | Kurchas | Onge | 0.0455 | 22.051 | 5844 | 5335 | 91780 |
| Kumhar | Jew_AP | Kurchas | Onge | 0.0487 | 21.961 | 5848 | 5305 | 91780 |
| Kumhar | Kathodi | Kurchas | Onge | 0.047 | 21.954 | 5854 | 5329 | 91780 |
| Kumhar | Kalash | Kurchas | Onge | 0.0441 | 21.899 | 5907 | 5408 | 91780 |
| Kumhar | Baniya | Kurchas | Onge | 0.0435 | 21.872 | 5859 | 5370 | 91780 |
| Kumhar | Makrani | Kurchas | Onge | 0.0399 | 21.832 | 5901 | 5448 | 91780 |
| Kumhar | GujaratiA | Kurchas | Onge | 0.0435 | 21.741 | 5884 | 5394 | 91780 |
| Kumhar | Kurmi_MP | Kurchas | Onge | 0.0454 | 21.684 | 5853 | 5344 | 91780 |
| Kumhar | Maratha | Kurchas | Onge | 0.0441 | 21.651 | 5843 | 5350 | 91780 |
| Kumhar | GujaratiB | Kurchas | Onge | 0.041 | 21.582 | 5851 | 5391 | 91780 |
| Kumhar | GujaratiD | Kurchas | Onge | 0.0415 | 21.581 | 5829 | 5364 | 91780 |
| Kumhar | Muthuraja | Kurchas | Onge | 0.0477 | 21.548 | 5868 | 5334 | 91780 |
| Kumhar | Dushadh | Kurchas | Onge | 0.0452 | 21.547 | 5854 | 5348 | 91780 |
| Kumhar | Muslim_Bihar | Kurchas | Onge | 0.0447 | 21.538 | 5870 | 5368 | 91780 |
| Kumhar | Coorghi | Kurchas | Onge | 0.041 | 21.535 | 5841 | 5380 | 91780 |
| Kumhar | Brahmin_Bhatt | Kurchas | Onge | 0.0458 | 21.514 | 5870 | 5355 | 91773 |
| Kumhar | Scheduled_Caste_Haryana | Kurchas | Onge | 0.0486 | 21.482 | 5876 | 5331 | 91780 |
| Kumhar | Meghawal | Kurchas | Onge | 0.0441 | 21.478 | 5870 | 5374 | 91780 |
| Kumhar | Relli | Kurchas | Onge | 0.0548 | 21.451 | 5938 | 5321 | 91751 |
| Kumhar | Sonkar | Kurchas | Onge | 0.0457 | 21.423 | 5848 | 5336 | 91779 |
| Kumhar | Backward_Caste | Kurchas | Onge | 0.0446 | 21.351 | 5863 | 5362 | 91780 |
| Kumhar | Jains | Kurchas | Onge | 0.0436 | 21.257 | 5859 | 5369 | 91780 |
| Kumhar | Gugavellalar | Kurchas | Onge | 0.0469 | 21.169 | 5847 | 5323 | 91780 |
| Kumhar | Chamar_Haryana | Kurchas | Onge | 0.043 | 21.145 | 5851 | 5369 | 91780 |
| Kumhar | Shani_Sc | Kurchas | Onge | 0.0473 | 21.144 | 5839 | 5312 | 91779 |
| Kumhar | Mahadeo_Koli | Kurchas | Onge | 0.0476 | 21.133 | 5858 | 5326 | 91780 |
| Kumhar | Kuruchiyan | Kurchas | Onge | 0.0426 | 21.079 | 5833 | 5356 | 91780 |
| Kumhar | Sch_caste_TN | Kurchas | Onge | 0.0464 | 21.009 | 5798 | 5284 | 90674 |
| Kumhar | Adiyan | Kurchas | Onge | 0.0583 | 20.978 | 5626 | 5006 | 86782 |
| Kumhar | Kalinga | Kurchas | Onge | 0.0455 | 20.83 | 5874 | 5362 | 91780 |
| Kumhar | Malaikuarvar | Kurchas | Onge | 0.0451 | 20.799 | 5851 | 5346 | 91779 |
| Kumhar | Dharikhar | Kurchas | Onge | 0.0479 | 20.692 | 5869 | 5332 | 91780 |
| Kumhar | Chipi | Kurchas | Onge | 0.0472 | 20.601 | 5879 | 5350 | 91780 |
| Kumhar | Brahmin_Nepal | Kurchas | Onge | 0.0455 | 20.521 | 5881 | 5369 | 91780 |
| Kumhar | Lingayath_TN | Kurchas | Onge | 0.0453 | 20.497 | 5842 | 5335 | 91780 |
| Kumhar | Siddi_Gujarat | Kurchas | Onge | 0.0504 | 20.489 | 5874 | 5311 | 91779 |
| Kumhar | Meddari | Kurchas | Onge | 0.0453 | 20.378 | 5835 | 5329 | 91780 |
| Kumhar | Malai_Kuravar | Kurchas | Onge | 0.0435 | 20.179 | 5823 | 5338 | 91780 |
| Kumhar | Pattapu_Kapu | Kurchas | Onge | 0.0434 | 20.132 | 5840 | 5355 | 91780 |
| Kumhar | Lingayath_Karnataka | Kurchas | Onge | 0.0444 | 20.132 | 5842 | 5345 | 91780 |
| Kumhar | Gujjar | Kurchas | Onge | 0.0436 | 20.051 | 5898 | 5405 | 91780 |
| Kumhar | Vedda | Kurchas | Onge | 0.0508 | 20.048 | 5888 | 5320 | 91780 |
| Kumhar | Gamit | Kurchas | Onge | 0.0466 | 20.037 | 5858 | 5337 | 91780 |
| Kumhar | Jew_Ashkenazi | Kurchas | Onge | 0.0448 | 19.963 | 5989 | 5475 | 91780 |
| Kumhar | Changpa | Kurchas | Onge | 0.0495 | 19.961 | 5852 | 5300 | 91780 |
| Kumhar | Chamar_UP | Kurchas | Onge | 0.0523 | 19.959 | 5909 | 5321 | 91774 |
| Kumhar | Pandit | Kurchas | Onge | 0.0423 | 19.91 | 5881 | 5403 | 91780 |
| Kumhar | Sonr | Kurchas | Onge | 0.0684 | 19.908 | 5615 | 4895 | 85176 |
| Kumhar | Gaud_Karnataka | Kurchas | Onge | 0.0428 | 19.9 | 5836 | 5357 | 91780 |
| Kumhar | Bhilala | Kurchas | Onge | 0.044 | 19.895 | 5833 | 5342 | 91780 |
| Kumhar | Tadvi | Kurchas | Onge | 0.0453 | 19.892 | 5841 | 5334 | 91780 |
| Kumhar | Baiswar | Kurchas | Onge | 0.0445 | 19.859 | 5854 | 5355 | 91780 |
| Kumhar | Silawat | Kurchas | Onge | 0.0443 | 19.759 | 5853 | 5356 | 91780 |
| Kumhar | Brahmin_Karnataka | Kurchas | Onge | 0.0413 | 19.744 | 5847 | 5384 | 91780 |
| Kumhar | Brahmin_Haryana | Kurchas | Onge | 0.0441 | 19.652 | 5882 | 5385 | 91780 |
| Kumhar | Bestha | Kurchas | Onge | 0.0444 | 19.645 | 5840 | 5344 | 91779 |
| Kumhar | Narikkuravar | Kurchas | Onge | 0.0453 | 19.609 | 5858 | 5351 | 91780 |
| Kumhar | Oddari | Kurchas | Onge | 0.0445 | 19.294 | 5846 | 5348 | 91780 |
| Kumhar | Velama | Kurchas | Onge | 0.0418 | 19.226 | 5844 | 5375 | 91780 |
| Kumhar | Ho_Jharkhand | Kurchas | Onge | 0.0649 | 19.146 | 5991 | 5261 | 91706 |
| Kumhar | Khatri | Kurchas | Onge | 0.0432 | 19.039 | 5881 | 5393 | 91780 |
| Kumhar | Vadde | Kurchas | Onge | 0.0467 | 18.842 | 5561 | 5064 | 86802 |
| Kumhar | Tanti | Kurchas | Onge | 0.0524 | 18.765 | 5894 | 5306 | 91780 |
| Kumhar | Yadav_UP | Kurchas | Onge | 0.0433 | 18.685 | 5864 | 5377 | 91780 |
| Kumhar | Rajput | Kurchas | Onge | 0.0445 | 18.684 | 5875 | 5375 | 91780 |
| Kumhar | Shia_Iranian_Hyderabad | Kurchas | Onge | 0.0431 | 18.567 | 5923 | 5434 | 91780 |
| Kumhar | Barela | Kurchas | Onge | 0.0481 | 18.51 | 5840 | 5304 | 91775 |
| Kumhar | Lohana | Kurchas | Onge | 0.0441 | 18.484 | 5902 | 5404 | 91780 |
| Kumhar | Sah_Obc | Kurchas | Onge | 0.0464 | 18.396 | 5867 | 5346 | 91777 |
| Kumhar | Pasi | Kurchas | Onge | 0.0458 | 18.318 | 5848 | 5336 | 91779 |
| Kumhar | Jain | Kurchas | Onge | 0.0412 | 18.164 | 5832 | 5370 | 91780 |
| Kumhar | Chamada | Kurchas | Onge | 0.0402 | 17.539 | 5807 | 5358 | 91780 |
| Kumhar | Kondaamari | Kurchas | Onge | 0.0591 | 17.454 | 5855 | 5202 | 90459 |
| Kumhar | Achary | Kurchas | Onge | 0.0483 | 17.298 | 5885 | 5342 | 91780 |
| Kumhar | Lohar | Kurchas | Onge | 0.0456 | 17.173 | 5866 | 5354 | 91758 |
| Kumhar | Dhokkali | Kurchas | Onge | 0.0457 | 16.999 | 5856 | 5344 | 91778 |
| Kumhar | Kissan | Kurchas | Onge | 0.0625 | 16.676 | 5939 | 5241 | 91732 |
| Kumhar | Budagajangam | Kurchas | Onge | 0.0406 | 15.642 | 5823 | 5369 | 91657 |
| Kumhar | Muslim_Jat | Kurchas | Onge | 0.0424 | 15.409 | 5890 | 5411 | 91779 |
| Kumhar | Dawoodi | Kurchas | Onge | 0.0415 | 15.227 | 5843 | 5378 | 91755 |
| Kumhar | Agamudayar | Kurchas | Onge | 0.0503 | 13.746 | 5787 | 5233 | 90277 |
| Kumhar | Padmashali | Kurchas | Onge | 0.0471 | 13.472 | 5861 | 5335 | 91718 |
| Kumhar | Gowli | Kurchas | Onge | 0.0467 | 13.26 | 5856 | 5333 | 91521 |
| Kumhar | Mudaliar | Kurchas | Onge | 0.0476 | 13.08 | 5464 | 4967 | 85326 |
| Kumhar | Tamta | Kurchas | Onge | 0.0468 | 13.042 | 5864 | 5340 | 91735 |
| Kumhar | Syed | Kurchas | Onge | 0.0451 | 12.879 | 5486 | 5012 | 85515 |
| Kumhar | Devendrakulathan | Kurchas | Onge | 0.0428 | 12.347 | 5603 | 5143 | 87777 |
| Kumhar | Malmi | Kurchas | Onge | 0.0437 | 12.214 | 5841 | 5352 | 91746 |
